# Weak Vestibular Response in Persistent Developmental Stuttering

**DOI:** 10.3389/fnint.2021.662127

**Published:** 2021-09-01

**Authors:** Max Gattie, Elena V. M. Lieven, Karolina Kluk

**Affiliations:** ^1^Manchester Centre for Audiology and Deafness (ManCAD), The University of Manchester, Manchester, United Kingdom; ^2^Child Study Centre, The University of Manchester, Manchester, United Kingdom; ^3^The ESRC International Centre for Language and Communicative Development (LuCiD), The University of Manchester, Manchester, United Kingdom

**Keywords:** stuttering, VEMP, vestibular, speech-motor control, own voice identification, speech perception

## Abstract

Vibrational energy created at the larynx during speech will deflect vestibular mechanoreceptors in humans (Todd et al., 2008; Curthoys, 2017; Curthoys et al., 2019). Vestibular-evoked myogenic potential (VEMP), an indirect measure of vestibular function, was assessed in 15 participants who stutter, with a non-stutter control group of 15 participants paired on age and sex. VEMP amplitude was 8.5 dB smaller in the stutter group than the non-stutter group (*p* = 0.035, 95% CI [−0.9, −16.1], *t* = −2.1, d = −0.8, conditional *R*^2^ = 0.88). The finding is subclinical as regards gravitoinertial function, and is interpreted with regard to speech-motor function in stuttering. There is overlap between brain areas receiving vestibular innervation, and brain areas identified as important in studies of persistent developmental stuttering. These include the auditory brainstem, cerebellar vermis, and the temporo-parietal junction. The finding supports the disruptive rhythm hypothesis (Howell et al., 1983; Howell, 2004) in which sensory inputs additional to own speech audition are fluency-enhancing when they coordinate with ongoing speech.

## Introduction

Persistent developmental stuttering manifests as prolongations or repetitions of speech sounds, or blocks to airflow, characteristically accompanied by increased tension in muscles of the face and articulatory system (Bloodstein et al., 2021). Behavioral manifestation is accompanied by differences in neurological activity and morphology by comparison with ordinarily fluent speakers (Budde et al., 2014; Belyk et al., 2015; Neef et al., 2015a; Etchell et al., 2017).

A consistent finding in stuttering research is that the amount of stuttering can be reduced with alterations to timing and/or audition during ongoing speech. Examples of fluency-inducing interventions for people who stutter include speaking with masking (Kern, 1932; Cherry et al., 1956), with a metronome (Barber, 1939; Fransella and Beech, 1965), in chorus with another speaker (Barber, 1940; Cherry et al., 1956), or in tandem with delayed (Neelly, 1961; Yates, 1963) and frequency-shifted (Howell et al., 1987) playback of ongoing speech. The findings are to a large degree captured by the disruptive rhythm hypothesis (Howell et al., 1983; Howell, 2004), which proposes that sensory inputs additional to own speech audition will be maximally fluency-enhancing when they coordinate with ongoing speech.

Research since the 1990s shows that the vestibular system in mammals responds to sonic and vibratory frequencies up to 1,000Hz, and may phase lock to higher frequencies (Rosengren and Colebatch, 2018; Curthoys et al., 2019). Vestibular sensitivity is considerably greater to vibrations conducted through the body than to sound waves in air (Welgampola et al., 2003), so much so that body-conducted vibration created by the act of speaking will deflect vestibular mechanoreceptors in humans (Todd et al., 2008; Curthoys, 2017; Curthoys et al., 2019). Electrophysiological responses of vestibular origin in humans are present at 70 dB above perceptual threshold for air-conducted stimuli, and 35 dB above perceptual threshold for body-conducted stimuli (McNerney and Burkard, 2011; includes adjustment for temporal integration). Thus, when referenced to a 60 dBA sound level typical of conversational speech, the indication is that air-conducted vestibular thresholds will be 10 dB above baseline and body-conducted vestibular thresholds 25 dB below baseline.

Deflection of vestibular mechanoreceptors by the vibrational energy created by speech sets off a chain of activity culminating in neural firing along the VIII cranial nerve. These neural firing patterns of vestibular origin will be coordinated with ongoing speech and, according to the disruptive rhythm hypothesis, will enhance fluency. Contrariwise, if neural firing patterns of vestibular origin are delayed or attenuated, dysfluency would be expected. This study was pre-registered (Gattie et al., 2019) with the hypothesis that vestibular-evoked myogenic potentials (VEMPs) in a stutter group would have significantly smaller amplitudes or significantly different latencies than in a non-stutter control group. Either result would support an interpretation in which the neural firing patterns arising from deflection of vestibular and cochlear mechanoreceptors combine differently between people who do and do not stutter.

## Materials and Methods

### Background

In addition to the corticopetal and corticofugal pathways typical to sensory systems, the vestibular system comprises reflexes causing body movements compensatory to changes in head position or body rotation. Examples include the vestibulo-collic reflex, which maintains balance, and the vestibulo-ocular reflex, which maintains direction of gaze (Beraneck et al., 2014). Automatic operation of reflex arcs via the brainstem (i.e., with no requisite cortical mediation) enables a faster motor response than would be possible if cortical involvement was necessary (Goldberg, 2012).

**FIGURE 1:**
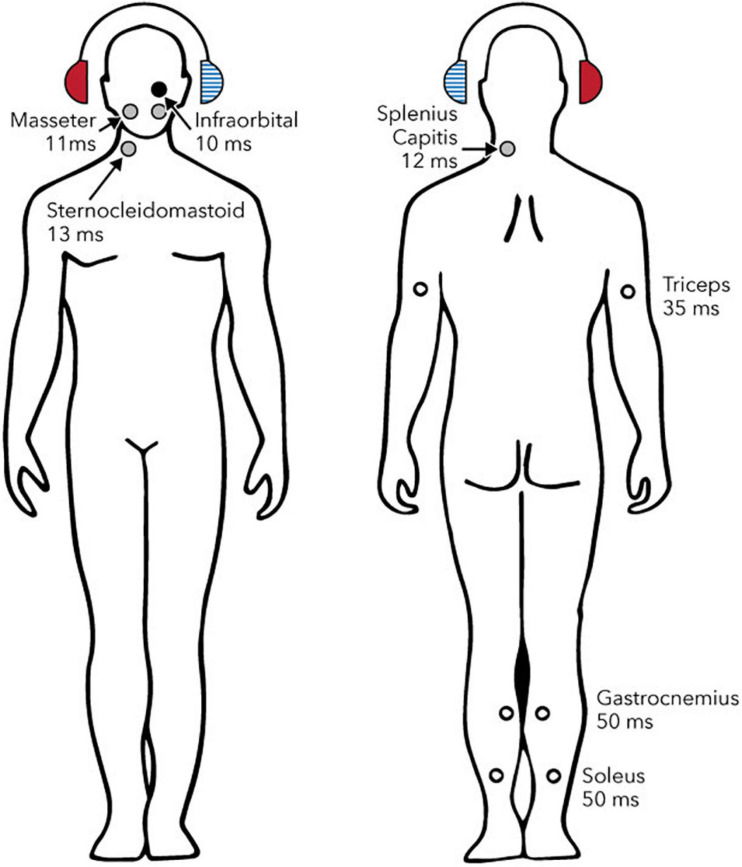
Reflexes evoked by sound or vibration in postural muscles. Circles show sites, laterality and approximate latencies based on air-conducted stimulation. The right ear (solid red headphone) is the stimulated side. Solid circles show reflexes whose polarity has been confirmed with intramuscular recordings (black: excitatory; grey: inhibitory). Open circles show reflexes whose polarity has either not been definitively determined (triceps and gastrocnemius) or is known to depend upon head position (soleus). Reproduced from Rosengren and Colebatch (2018), see original for references to supporting studies. Creative Commons Attribution License (CC-BY).

**FIGURE 2:**
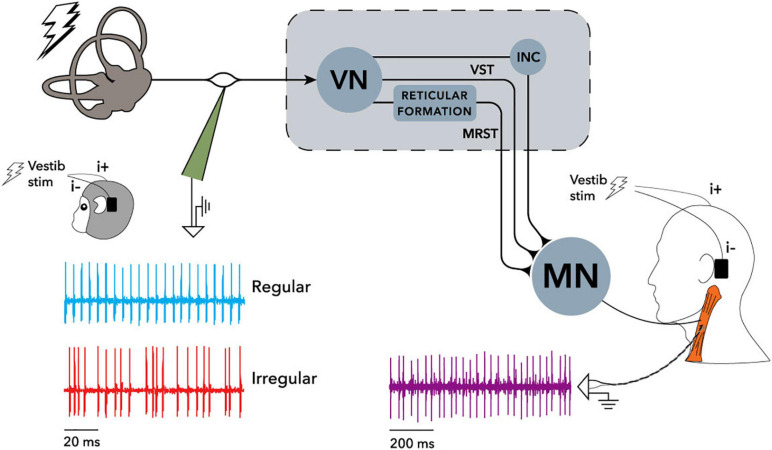
Recording arrangement and neural pathways of the electrically evoked vestibulo-collic reflex in monkey and human, reproduced from Forbes et al. (2020). Single motor unit recordings were made from the sternocleidomastoid muscle (SCM) using irregular stimuli, and sine wave stimuli at frequencies up to 300 Hz. Cervical motor unit activity in both human and monkey was modulated by the stimuli. Recording of vestibular afferents in the monkey only showed similar modulation. See Forbes et al. (2020) for detail of filtering and phase locking effects. When evaluated using surface electrodes and sound or vibration stimuli, inhibition of SCM spindles can be measured as the cervical vestibular evoked myogenic potential (VEMP) which is the subject of the current study. INC, interstitial nucleus of Cajal; MN, motoneurons; MRST, medial reticulospinal tract; VN, vestibular nuclei; VST, vestibulospinal tract. Creative Commons Attribution License (CC-BY).

Figure 1 shows reflexes identified in postural muscles. Figure 2 shows pathways for the vestibulo-collic reflex. Modelling of the vestibulo-collic reflex, including appraisal of relative contributions from saccule, utricle and vestibular canals, and exact trajectory through vestibular nuclei, remains ongoing (Forbes et al., 2013). The vestibulo-collic reflex might in principle be recorded from any neck muscle (Forbes et al., 2018). A short latency fragment of the vestibulo-collic reflex, referred to as a cervical VEMP, is frequently recorded using surface electrodes over the sternocleidomastoid muscle (SCM) (Goldberg and Cullen, 2011).

The VEMP measures a short inhibition of tonic activity in the SCM (Corneil and Camp, 2018; Rosengren and Colebatch, 2018). It is a large response having a characteristic peak (p1) and trough (n1). Measures of interest include the difference in amplitude (p1-n1 amplitude) and time (p1-n1 latency) between the characteristic peak and trough. In modelling studies, the VEMP represents a superposition of motor unit action potentials occurring at irregular time intervals (Wit and Kingma, 2006), with generation of motor unit action potentials being inhibited following presentation of sound or vibration. The VEMP can be described by two mathematical functions: one specifies the mean number of motor unit action potentials per unit of time, and the other describes the time course of an individual motor unit action potential (Lütkenhöner, 2019). As such, the VEMP does not correspond directly to neural firing rates of interest in the current study (i.e., those along the VIII cranial nerve or within vestibular nuclei). In this way, interpretation of data is disanalogous to experiments whose outcome measures do directly correspond to neural firing rates of interest (e.g., many study designs using single cell recordings, electrocorticography or electroencephalography).

Vestibular-evoked myogenic potentials do not provide a complete appraisal of the vestibular system, and find clinical application as part of a neuro-otological test battery. Cervical VEMPs are used clinically to identify acute vestibular syndrome, episodic vertigo, chronic dizziness or imbalance, and superior canal dehiscence and third window syndromes (Rosengren et al., 2019).

### Participants

This was a case control study, with 15 participants who stutter and a non-stutter group of 15 paired controls. All participants had normal hearing as assessed by otoscopy, tympanometry and pure tone audiometry. Stuttering was assessed using the SSI-4 (Riley, 2009). Non-stutter control participants had SSI-4 scores lower than 10, whilst participants who stutter had SSI-4 scores between 18 and 39 (a range from “mild” to “very severe” according to the SSI-4).

Stutter and non-stutter groups were paired on sex, and to within 0.05 years (SD 1.05) on age in aggregate. Of the 15 non-stutter controls, the seven participants aged younger than 21 years were selected from a normative sample of 48 undergraduate students (Gattie et al., in preparation). VEMP response amplitudes in these controls are representative of the normative sample of 48, rather than a normative sample of seven as would have been the case if controls aged younger than 21 years had been sampled randomly from the general population. Full details of screening and pairing are available in the Supplementary Material.

Prior to any testing, all participants gave written informed consent according to the Declaration of Helsinki. The University of Manchester Ethics Committee approved the study.

### Electromyography

Vestibular-evoked myogenic potentials were recorded on an Eclipse EP25 system (Interacoustics AS, Assens, Denmark). Disposable non-metallic silver chloride electrodes were used (type M0835, Biosense Medical, Essex, United Kingdom). Skin was prepared with NuPrep^®^ (Weaver and Company, CO, United States) prior to electrode attachment using Ten20^®^ conductive paste (Weaver and Company, CO, United States). Electrode impedances were maintained below 3 kΩ. An active electrode was placed over the SCM on the right hand side, with reference and ground electrodes on the upper sternum and nasion, respectively.

The stimulus was a 500 Hz sinusoidal carrier with rectangular windowing generated by the Eclipse. This frequency is found to be optimal for VEMP testing (Rosengren et al., 2010; Papathanasiou et al., 2014). The rise/fall time of zero, and plateau time of 2 ms, gave characteristics intermediate between a tone burst and a click (Laukli and Burkard, 2015). Stimuli were delivered at a rate of 5.1 per second through a B81 bone conductor (Radioear, MN, United States), positioned on the mastoid bone behind the right ear. The bone conductor was calibrated with a Model 4930 artificial mastoid and 2250 Investigator (Brüel and Kjaer, Naerum, Denmark), and an Agilent 54621A 2-Channel Oscilloscope (Keysight, CA, United States). Calibrations were based on the artificial mastoid having a reference equivalent threshold force level re 1 μN of 40.2 dB for 500 Hz. Interacoustics provide a correction factor of 69.5 dB for peSPL to nHL conversion of a 2-2-2 500 Hz tone burst. This correction factor was applied to the 0-1-0 500 Hz tone burst, with bone conduction levels accordingly reported in dB HL. Thus, stimulus levels in this report are calculated as they apply to the cochlea, rather than the vestibular system. More precisely, the stimulus levels describe a body conducted equivalent to standardised sound pressure levels in the ear canal. Maximum stimulus level was set at 40 dB HL, since sine waves with amplitude above 40 dB HL displayed clipped on the oscilloscope.

The electromyography (EMG) signal was amplified and band-pass filtered prior to sampling on the Eclipse system, using the Interacoustics research license. Low pass was a digital FIR filter of 102nd order at 1500 Hz, and high pass was a 10 Hz analog Butterworth filter of 1st order at 6 dB per octave. Sample rate was 3 kHz.

### Procedure

**FIGURE 3:**
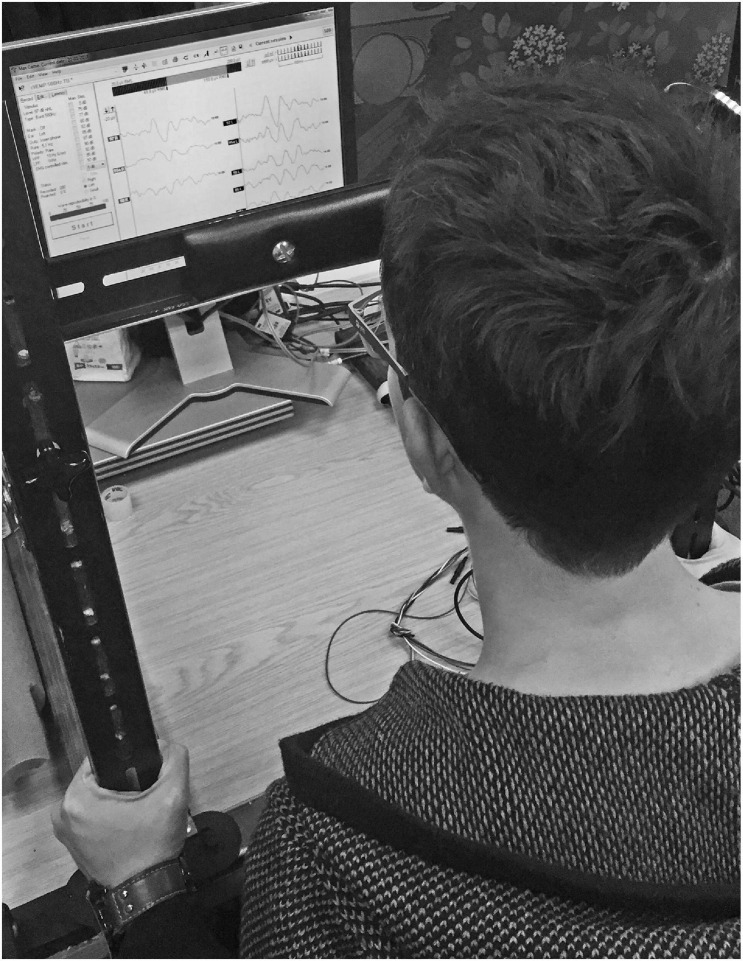
Custom head bar. Participants were instructed to push against a padded bar using the forehead, such that sternocleidomastoid tension was maintained as close as possible to 50 μV RMS throughout testing. Biofeedback in the Eclipse clinical software enabled participants to monitor sternocleidomastoid tension.

Participants were seated with the forehead resting against a padded bar, using apparatus specially constructed for this experiment (Figure 3). Participants were instructed to push their heads against the padded bar such that they would maintain an EMG biofeedback target as close as possible to 50 μV root mean square (RMS) throughout testing. If the background EMG was lower than 50 μV RMS, the stimulus would stop playing and participants were instructed to push harder. Participants were asked to push no harder than they needed to, and would rarely attempt to do so. The importance of maintaining a constant background EMG was relayed to participants, and the experimenter monitored background EMG throughout.

Eclipse recordings followed the Interacoustics recommended procedure for VEMPs, including rejection of epochs having peak or trough amplitudes with magnitude larger than ±800 μV. A software feature compensated for rejected epochs such that the averaged response to exactly 300 epochs was recorded for every stimulus level tested. Such averages of 300 epochs will be referred to henceforth as “sequences.” The initial sequence was recorded with a stimulus level of 40 dB HL, with further sequences recorded with stimulus level descending in 2 dB steps until 34 dB HL or until the averaged VEMP trace summarising the sequence was comparable to background noise, whichever came soonest. Comparison of the averaged VEMP trace to background noise was made by the experimenter using the EP25 clinical software. A second series of recordings was initiated at 39 dB HL, with stimulus level descending in 2 dB steps until 35 dB HL or until the averaged VEMP trace summarising the sequence was comparable to background noise, whichever came soonest. The collection procedure was explained to participants, who could watch their averaged VEMP trace being calculated in real time by the EP25 software on a computer screen. If the participant was willing (e.g., if they had no time constraints) and if participants had shown a response at 34 dB HL, further sequences were recorded at stimulus levels below 34 dB HL. Sessions ended with repeat recording of a sequence using the maximum 40 dB HL stimulus level.

### Data Processing

Raw data were processed using custom scripts in MATLAB 2019a (The MathWorks, Inc., Natick, MA, United States). Response amplitudes were transformed into a dimensionless ratio by normalising per participant. For each participant, a pre-stimulus interval of 18 ms was extracted from a mean of the EMG waveforms from the first six sequences of 300 presentations recorded (i.e., it was a pre-stimulus mean of the first 1800 presentations recorded). The RMS of this per participant pre-stimulus mean was assigned as a background EMG tension per participant. Finally, all waveforms for a participant were normalised by dividing them by the background EMG tension per participant.

This normalisation procedure is in principle not necessary, since background EMG tension is already tightly controlled at a target of 50 μV per participant using the head bar. However, the normalisation will account for any small per participant variation in background EMG tension. Normalisation uses the maximum pre-stimulus data available for every participant (1800 presentations), minimising the presence of random noise per participant in the pre-stimulus RMS background EMG tension. This procedure is preferable to, for example, per sequence normalisation based on pre-stimulus RMS for each sequence of 300 presentations. Per sequence normalisation would introduce noise to data because random fluctuation in pre-stimulus RMS per sequence (i.e., random in addition to any actual change in sternocleidomastoid tension) would affect VEMP amplitudes randomly on a per sequence basis, thereby affecting within participant comparisons. Between participant comparisons will use linear mixed-effects regression analysis, which depends on an accurate within participant measure of VEMP amplitude growth with stimulus level. As such, preserving within participant comparisons as accurately as possible – so, identically to the raw data with the normalisation procedures used in this study – is optimal for linear mixed-effects regression analysis.

**FIGURE 4:**
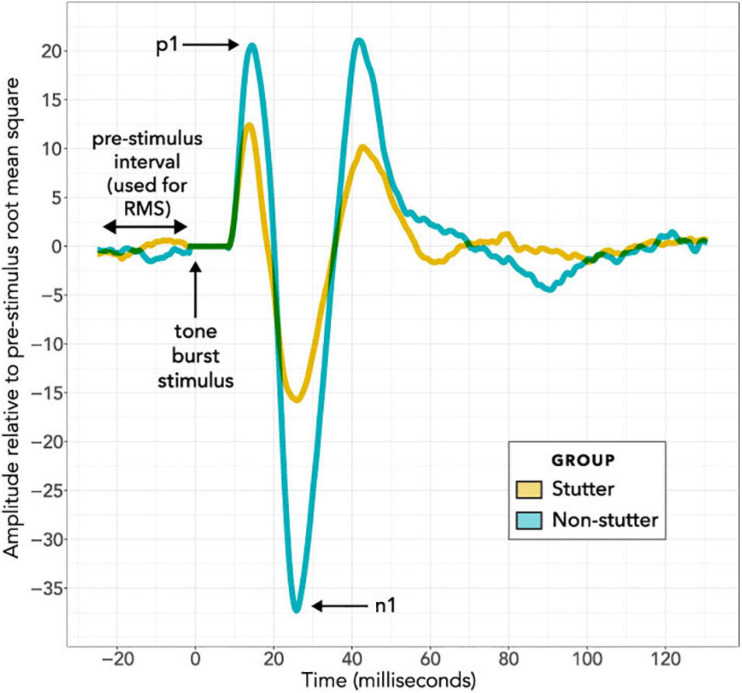
Grand Average VEMP wave forms at the maximum 40 dB HL stimulus level. The horizontal axis shows the time course of each epoch in milliseconds, with the stimulus always presented at time zero during an epoch. The 8 ms interval immediately after stimulus presentation is adjusted to have an amplitude of zero for all recordings, to remove stimulus artefact from the bone conductor. The vertical axis shows response amplitude. Wave forms in this figure have been averaged per participant and per group. On a per participant basis, the 300 epochs per stimulus level per participant were averaged together; these averages of 300 epochs (see the “Procedure” and “Data Processing” sections) are referred to as a “sequence”. Normalisation was then carried out on a per participant basis, and is in addition to the tight control of background electromyographic tension (target of 50 μV for all participants) using a custom head bar and biofeedback. In the normalisation routine, the VEMP amplitude of the wave form in microvolts was divided by the root mean square VEMP amplitude in microvolts of an 18 ms pre-stimulus interval. VEMP amplitudes are thus provided in dimensionless units. In the per group averaging to create the grand averages shown in this figure, all normalised sequences at 40 dB HL have been averaged together on a group basis for either the stutter group or the non-stutter control group.

Figure 4 shows VEMP grand averages for stutter and non-stutter groups at the maximum 40 dB HL stimulus level. Peaks per sequence per participant were identified using the “findpeaks” algorithm in the MATLAB Signal Processing Toolbox. Waveforms were inverted to find troughs. Initially peaks and troughs were appraised for the first 40 dB HL sequence per participant. This was done by first identifying troughs for the entire 40 dB HL sequence, and then identifying the most prominent trough (prominence as defined in the findpeaks algorithm) between 15 and 37 ms as n1. Next, peaks were identified for the entire 40 dB HL trace. Peaks earlier than 8 ms, and later than n1, were discarded. Remaining peaks were ranked. Firstly, the three most prominent peaks were awarded 5, 4, and 3 points in order of prominence. Secondly, the same three most prominent peaks were weighted based on their prominence compared to the most prominent peak: 3 points awarded for greater than or equal to two thirds; 2 points for greater than one third and less than two thirds; and 1 point otherwise. Thirdly, the five peaks having the smallest time difference from n1 were awarded points from 5 to 1 in a hierarchy with more points for smaller time difference. Finally, all of the points were summed. The peak with the greatest number of points was identified as p1. Ties were decided in favor of the peak with smaller time difference from n1.

Peaks and troughs for other stimulus levels were identified in a similar manner to the process just described for the initial 40 dB HL sequence, except that the trough from the initial 40 dB HL sequence was used as an anchor for trough detection for remaining sequences on a per participant basis. Peaks and troughs were rejected (the script returned an empty result) if the p1-n1 amplitude was less than 1.65 times the pre-stimulus RMS for the sequence of 300 repetitions being evaluated.

The script was checked through visual inspection of waveforms for the entire data set collected. This was an iterative procedure, with the script run several times using adjustments to some of the parameters described. Visual inspection showed that the final script identified peaks and troughs with a high degree of fidelity. Identification by the script was final – no data points were removed or adjusted manually.

Data were transformed to a response level (RL) scale by taking the log of p1-n1 amplitude as follows:


$$\mathrm{p1}-\mathrm{n1}\text{amp}\left(\mathrm{dB}\mathrm{RL}\right)=20\times \log_{10}\left(\begin{array}{c} \mathrm{p1}-\mathrm{n1}\text{amp}\left(\mu \text{V}\right)\\ \bar{\text{prestimulus}\text{RMS}\left(\mu V\right)} \end{array}\right)-20$$

Zero dB RL denotes a projected VEMP threshold (this is not the same as VEMP thresholds in clinical procedure; see note at Figure 11). The transformation is analogous to that for the dB SPL scale widely used for sound pressure levels (and its frequency-adjusted HL variant), in which a 10 dB increase approximates a perceptual doubling.

### Confounders in VEMP Measurement

This section describes precautions taken to minimise potential confounders in VEMP measurement. The precautions predominantly address measurement of VEMP p1-n1 amplitude, but will also increase accuracy when measuring VEMP p1-n1 latency.

### Stimulus Level

VEMP p1-n1 amplitude is expected to increase with stimulus level (Todd et al., 2008). Linear mixed-effects regression analysis takes advantage of this relationship, with between group comparisons based on VEMP growth rate.

### Neck Tension

Tension in the SCM must be greater than at resting state in order to record a cervical VEMP. However, VEMP p1-n1 amplitude increases with SCM tension (Ochi et al., 2001). Accordingly, variation in SCM tension was limited, to prevent it acting as a confounder. This was done by asking participants to maintain a constant biofeedback target whilst pushing against a padded head bar (Figure 3).

Additional measures were taken to ensure that SCM tension did not act as a confounder. Pre-stimulus SCM tension was measured so that it could, if necessary, be included as a covariate during analysis. To ensure that fatigue could not be a factor, duration of testing was also assessed as a covariate.

### Age

Participants were paired on age to control for a decrease in VEMP p1-n1 amplitude with age (Nguyen et al., 2010; Colebatch et al., 2013).

### Crossed Response

Cervical VEMPs are predominantly ipsilateral, but may sometimes have a contralateral component (Colebatch and Rothwell, 2004; Ashford et al., 2016). Use of binaural stimuli limited variation due to any between participant difference in the extent of contralateral activity, because ipsilateral and contralateral components of the VEMP from each ear were present at both SCM muscles. The arrangement is imperfect, because the mastoid placement for the bone conductor introduces an asymmetry, with approximately 3–5 dB intracranial attenuation for the 500 Hz tone burst used (Stenfelt, 2012). However, this asymmetry in body-conducted stimulation is consistent per participant.

### Sternocleidomastoid Physiology

Sternocleidomastoid muscle size and subcutaneous fat are likely to influence VEMP amplitude (Chang et al., 2007; Bartuzi et al., 2010). The effect was not appraised, although it was minimised by the normalisation procedure, the pairing on age and sex, and the use of amplitude growth parameters for between group comparisons.

### Blood Flow

Blood has electromagnetic properties (Beving et al., 1994; Abdalla, 2011) meaning electromagnetic field variations due to blood flow will add noise to EMG recordings. The active electrode placement for cervical VEMPs, directly above the carotid artery, suggests that measurement of cervical VEMPs will be affected by blood flow. This is mitigated by the large size of the cervical VEMP response. Stimuli were delivered at a rate of 5.1 per second, whilst resting state pulse rates are approximately one per second. As a result, variations in the EMG recording due to carotid artery blood flow will largely cancel out over the approximately 1 min recording time, such that noise due to blood flow is minimal.

### Statistical Model

**FIGURE 5:**
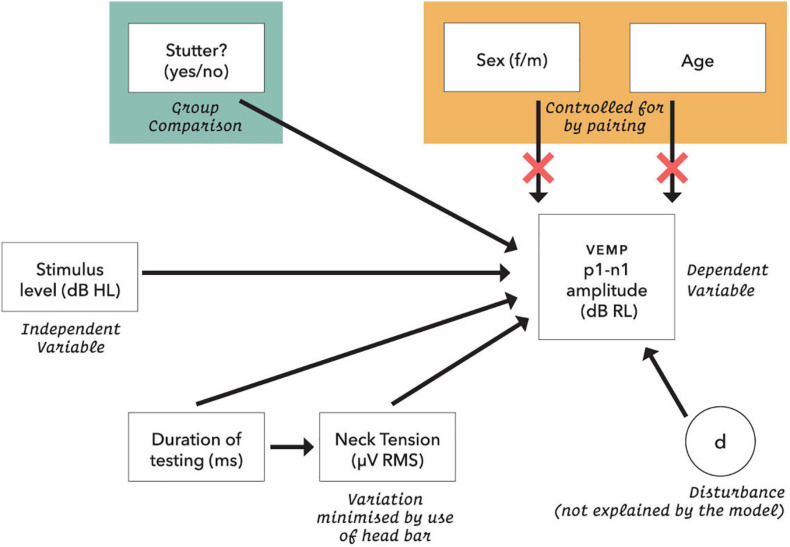
Initial statistical model for VEMP p1-n1 response amplitude. Neck tension was a root mean square of the pre-stimulus VEMP p1-n1 amplitude based on each presentation sequence of 300 stimulus repetitions. Age was calculated in days at the time of testing. The dB RL units used for vestibular response are a log transformation of the VEMP p1-n1 amplitude, such that zero dB RL corresponds to vestibular threshold (although, see note in Figure 11). Possible disturbances include neck size, pulse rate and crossed response.

The initial statistical model for VEMP p1-n1 amplitude is shown in Figure 5. Preliminary analysis with data from 48 control participants (Gattie et al., in preparation) eliminated neck tension and duration of testing as confounders for amplitude or latency measures. It also showed that VEMP p1-n1 latency is independent of stimulus level. This simplifies the latency model, because the only remaining predictor is whether or not a participant stutters.

For VEMP p1-n1 amplitudes, linear mixed-effects regression modelling (Winter, 2019) follows the form:


$$\text{VEMP}\mathrm{p1}-\mathrm{n1}\text{amplitude}=\beta_{0_{j}}+\beta_{1}\times \text{stutter}+\varepsilon$$

Where VEMP p1-n1 amplitude is conditioned on whether or not participants stutter, with ß_0_ as intercept (varies with participant, j) and ß_1_ as a fixed slope of increase in VEMP p1-n1 amplitude with stimulus level. Varying slope models were also appraised (see Supplementary Material). Statistical analysis was conducted with the lme4 package (Bates et al., 2015) in R (R Development Core Team, 2020). Effect size (Cohen’s d) was calculated from mixed model t statistics with the EMAtools package for R, version 0.1.3 (R Foundation). Conditional R^2^ was calculated according to Nakagawa et al. (2017) using the MuMIn package, version 1.43.17 (R Foundation).

## Results

### VEMP p1-n1 Amplitude

**FIGURE 6:**
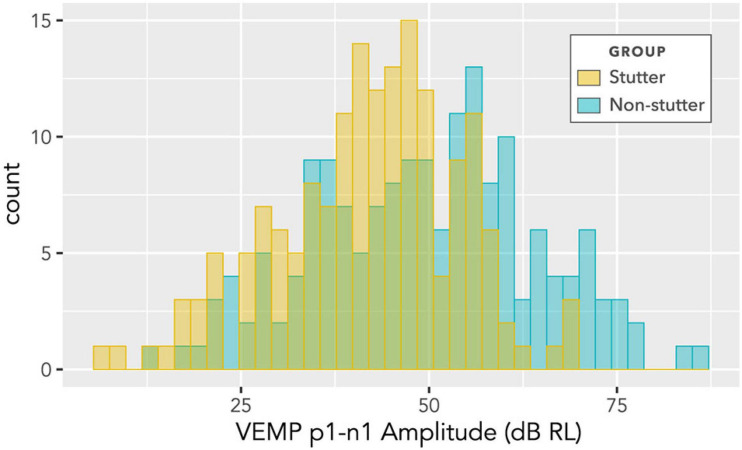
Histogram of VEMP p1-n1 amplitudes for stutter and control groups. The histogram does not show detail of participant or stimulus level, and contains repeated measurements for the two groups of 15 participants per group. As such, it suggests shape of distribution and direction of group difference, but is not appropriate for statistical comparison (statistical comparison is by linear mixed-effects regression modelling).

The histogram in Figure 6 shows counts of VEMP p1-n1 amplitude measurements sorted into stutter or non-stutter groups. The histogram does not show detail of participant or stimulus level. Since the histogram contains repeated measurements, it is not appropriate for statistical comparisons. However, presentation count was approximately equal per participant, and over approximately the same stimulus range, meaning that the histogram gives an indication of distribution for each group. Both the stutter and non-stutter groups appear to have a normal distribution, and there is suggestion of a difference between the means of the distributions.

**FIGURE 7:**
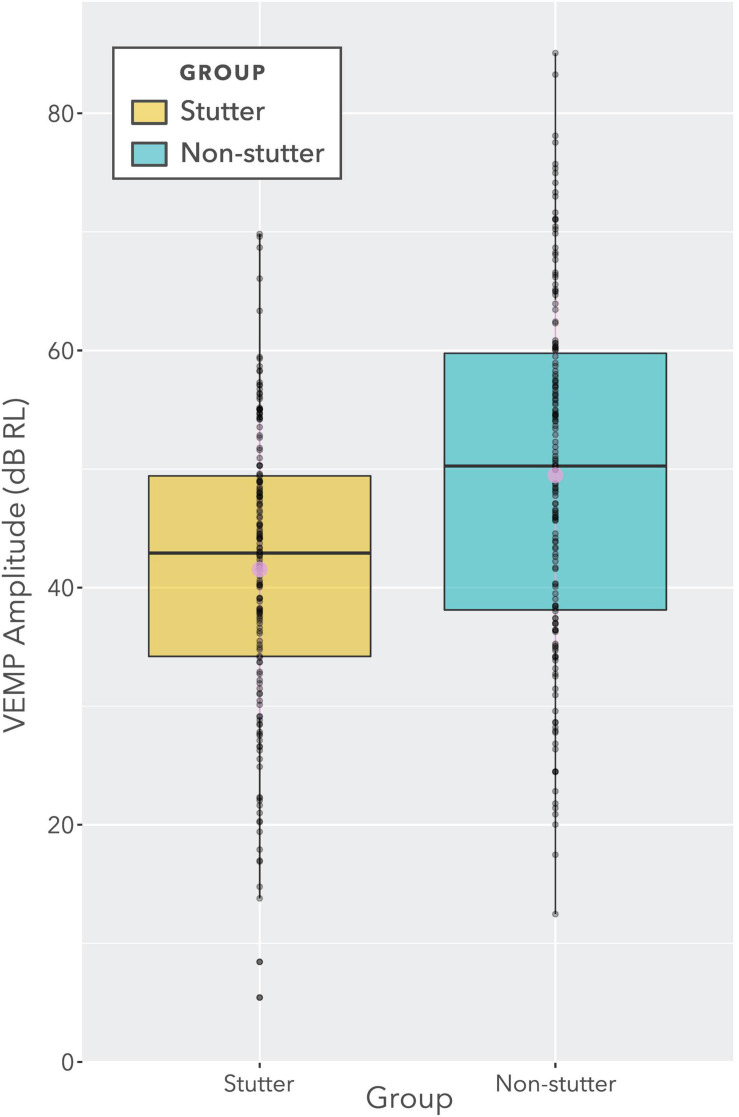
Boxplot showing VEMP p1-n1 amplitudes for stutter and non-stutter groups collapsed across stimulus level (i.e., identical data to Figure 6). Log transformation on the ordinate is such that a doubling of the VEMP p1-n1 amplitude in normalised microvolts (i.e., with unity RMS background) corresponds to a 6 dB increase. The two slightly larger circles near the medians denote means. The ratio of difference between medians to overall spread (i.e., to the difference between the lower quartile for the stutter group and the upper quartile for the non-stutter group) is approximately 30%. However, this data presentation is for illustration purposes only. The data contain repeat readings with asymmetries between groups. The actual statistical analysis is via linear mixed-effects regression modelling, and is described in the sections “Statistical Model” and “VEMP p1-n1 Amplitude”.

**FIGURE 8:**
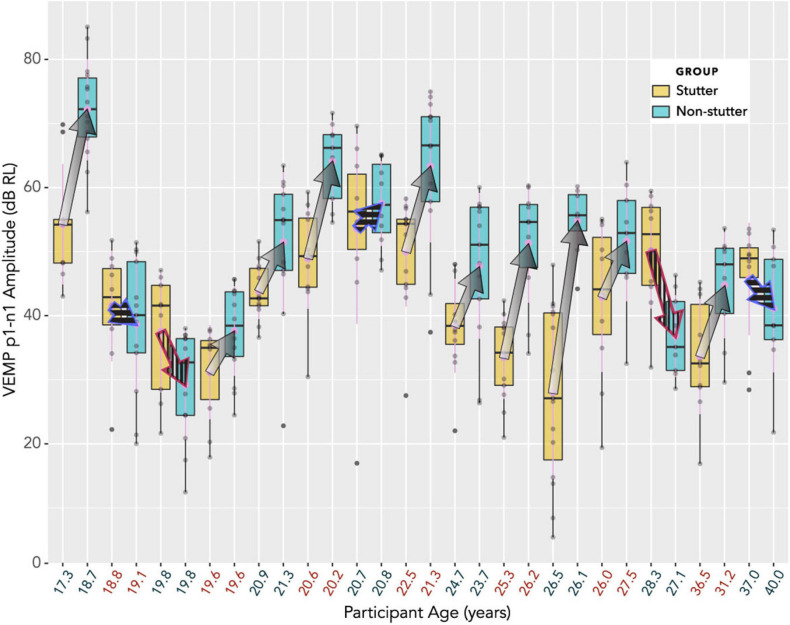
Box plots showing distributions of VEMP p1-n1 amplitude, with participants who stutter and paired non-stutter control participants arranged adjacently in order of age. The box plot does not show detail of stimulus level (although, larger VEMP p1-n1 amplitude almost invariably corresponds to higher stimulus level). Log transformation on the ordinate is such that a 6 dB increase corresponds to a doubling of the VEMP p1-n1 amplitude in normalised microvolts (i.e., with unity RMS background). Arrows link the mean VEMP p1-n1 amplitudes of participants who stutter with those of their paired controls. The control participant without stuttering is always shown at the point of the arrow, whilst the participant with stuttering is where fletching would appear. In 10 cases (arrows with gradients) VEMP p1-n1 amplitudes are overall markedly higher for non-stutter than stutter participants. In three cases (blue outline arrows with horizontal stripes) there is a partial overlap, which will be evaluated in the statistical analysis. In two cases (red outline arrows with vertical stripes) VEMP p1-n1 amplitudes are overall clearly higher for the stutter than the non-stutter participants. These two participants who stutter differed from the remaining 13 in the stutter group (one had a possible psychogenic onset, the other had both cluttering and stuttering). All 15 participants in the stutter group, along with the 15 control participants in the non-stutter group, were included in the linear mixed-effects regression analysis.

The box plot in Figure 7 provides an alternative view of the data in Figure 6. It should be compared with Figure 8, which shows per participant distributions of VEMP p1-n1 amplitude, with participants who stutter and paired non-stutter controls arranged adjacently in order of age. Box plots do not show detail of stimulus level. Figure 8 shows that for 10 of the 15 pairs, VEMP p1-n1 amplitudes are overall markedly higher for the non-stutter than the stutter participant. In 3 of the 15 pairs, there is a partial overlap, which will be evaluated through linear mixed-effects regression modelling. In two cases, VEMP p1-n1 amplitudes are overall clearly higher for the stutter than the non-stutter participant. However, stuttering in these two participants differed from the others in the stutter group. One participant had both cluttering and stuttering, whilst stuttering in the other had a possible psychogenic rather than developmental origin (see Supplementary Material). Data from both participants and their pairs were retained in the statistical analysis.

**FIGURE 9:**
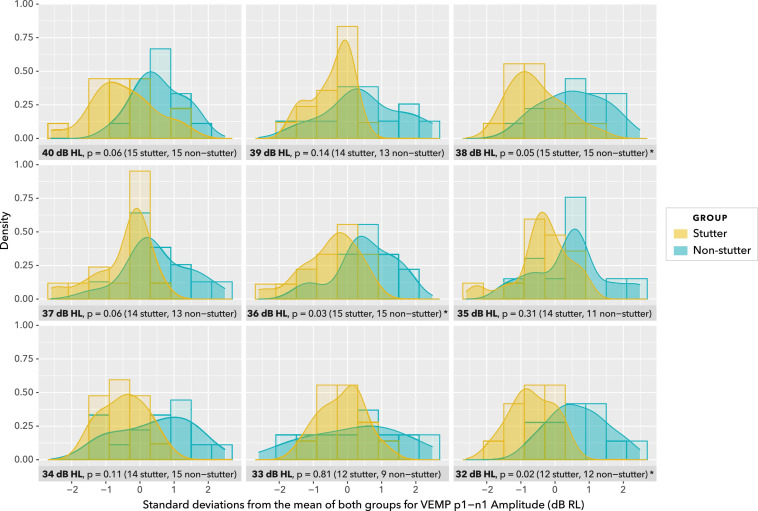
Density plots at stimulus levels between 40 dB HL and 32 dB HL. Histograms are shown in the background. Uncorrected *t*-tests show group differences at *p* ≤ 0.05 for 38, 36, and 32 dB HL, and *p* = 0.06 for 40 and 37 dB HL. Group sizes are unbalanced at 35 and 33 dB HL. This view of data with uncorrected *t*-tests is for illustration purposes only. Repeated measures at the same stimulus level are excluded, as are data at stimulus levels below 32 dB HL, and no account is made of trends in participants across stimulus levels. The actual statistical analysis is by linear mixed-effects regression modelling, and is described in the sections “Statistical Model” and “VEMP p1-n1 Amplitude.”

Density plots in Figure 9 provide a view of the data without detail of participants, but with detail of stimulus level. As such, they are complementary to the box plots in Figure 8. Uncorrected *t*-tests show group differences at or near an alpha level of 0.05 for five of the nine stimulus levels shown. However, such *t*-tests do not accurately summarise the data. Repeated measures at the same stimulus level are excluded from Figure 9 and from *t*-tests, as are data at stimulus levels below 32 dB HL, and no account is made of trends in individual participants across stimulus levels.

Pre-registration specified use of linear mixed-effects regression analysis. A random intercepts model gives the statistically significant result that the stutter group has a VEMP p1-n1 amplitude 8.5 dB smaller than the non-stutter group for the range of stimulus levels tested (*p* = 0.035, 95% CI [−0.9, −16.1], Chi-Squared (1) = 4.44, *d* = −0.8, conditional *R*^2^ = 0.88).

**FIGURE 10:**
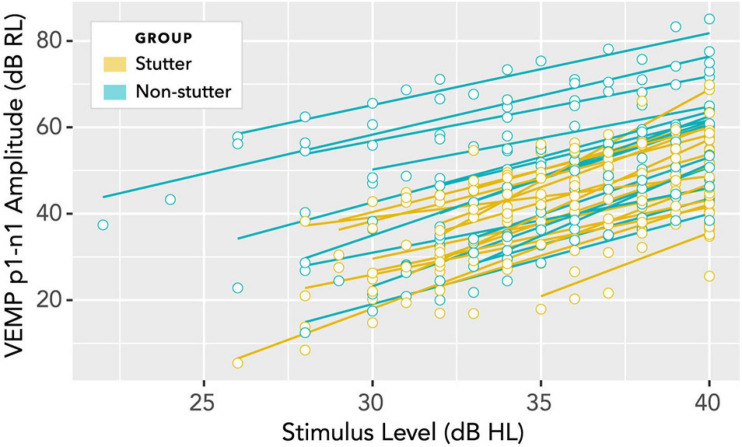
Per participant slopes of stimulus level (dB HL) versus VEMP p1-n1 amplitude (dB RL). Log transformation on the ordinate is such that a 6 dB increase corresponds to a doubling of the VEMP p1-n1 amplitude in normalised microvolts (i.e., with unity RMS background). A fixed slope, varying intercept model is supported if the least squares fit lines shown in this diagram are considered approximately parallel. Analyses of varying slope linear mixed models for these data are available in the Supplementary Material.

**FIGURE 11:**
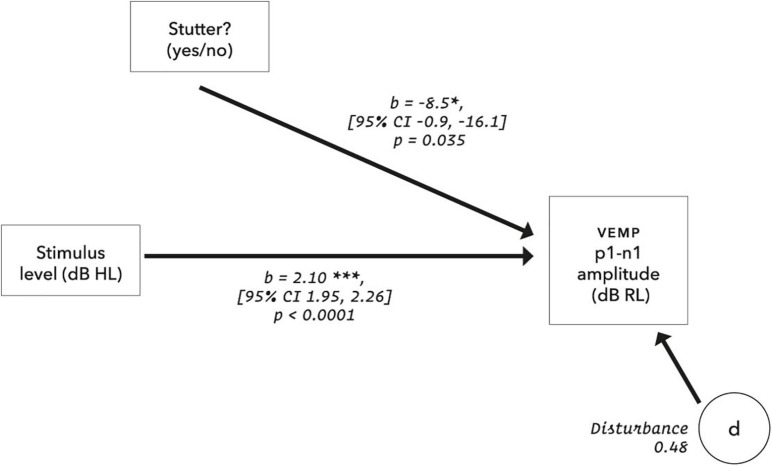
Final model for VEMP amplitude. The disturbance represents influences other than those measured in the model, and is the square root of (1 – *R*^2^), where the conditional R^2^ is calculated according to Nakagawa et al. (2017) using the MuMIn package (version 1.43.17). For the control group, using the calibrations and data transformations in this report, the *y*-axis intercept is −24.9 dB RL (95% CI [−32.6, −17.2]). The suggestion is of VEMP thresholds at 20.3 dB HL for the stutter group and 11.8 dB HL for the non-stutter group. However, VEMP thresholds projected in this way (extrapolation to 0 dB RL) assume a linear relationship between stimulus level and VEMP amplitude over a wider range of stimulus levels than was tested in this study. Such projections are dissimilar to VEMP thresholds evaluated by clinical search procedures (e.g., as per British Society of Audiology, 2012). Clinical VEMP thresholds refer to the smallest VEMP p1-n1 amplitudes which can be recorded against electromyographic background in a particular laboratory, and are used for differential diagnosis as part of a test battery (Rosengren et al., 2019).

In linear mixed-effects regression modelling, there is a trade-off between greater possibility of type I error when data from all participants are assigned the same slope but can have different intercepts, versus lower statistical power when both slope and intercept can vary with data per participant (Barr et al., 2013; Matuschek et al., 2017). Analysis of a wider range of models, including random slopes, is detailed in the Supplementary Material, along with an analysis of pre-stimulus RMS background EMG tension. A convergence warning with varying slopes can be removed by removing outlying data. All fixed and varying slope models evaluated give the result that VEMP p1-n1 amplitude is between 7.9 and 8.7 dB RL smaller in the stutter group than the non-stutter group, with *p*-values between 0.021 and 0.049. Slopes per participant are shown in Figure 10. It was because the slopes in Figure 10 are approximately parallel that the fixed slope, random intercepts model was preferred. The final model for VEMP amplitude is shown in Figure 11.

The study had a pilot, which was reanalyzed using the scripts developed for this main report. Comparison of 5 participants who stutter with matched controls gives a result similar to the main report, with VEMP p1-n1 amplitude 10.1 dB smaller in the stutter than the non-stutter group (*p* = 0.044, 95% CI [−1.3, −18.9]). The pilot study is described in more detail in the Supplementary Material.

### VEMP p1-n1 Latency

**FIGURE 12:**
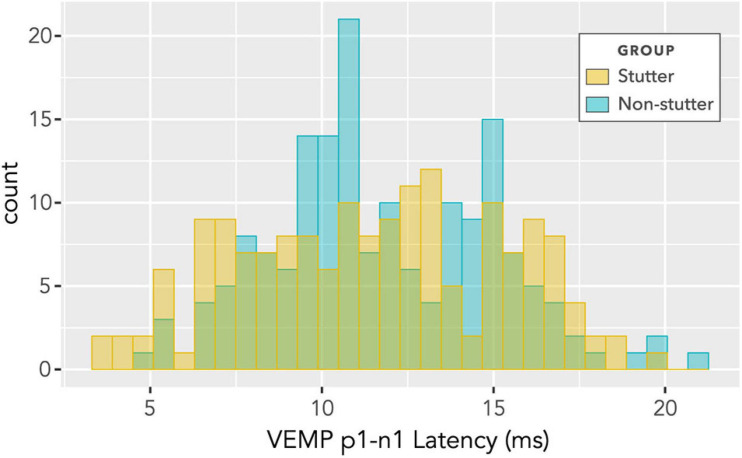
Histogram of VEMP p1-n1 latencies for stutter and control groups. The histogram does not show detail of participant or stimulus level, and contains repeated measurements for the two groups of 15 participants per group. As such, it suggests shape of distribution and direction of group difference, but is not appropriate for statistical comparison (statistical comparison is by linear mixed-effects regression modelling).

**FIGURE 13:**
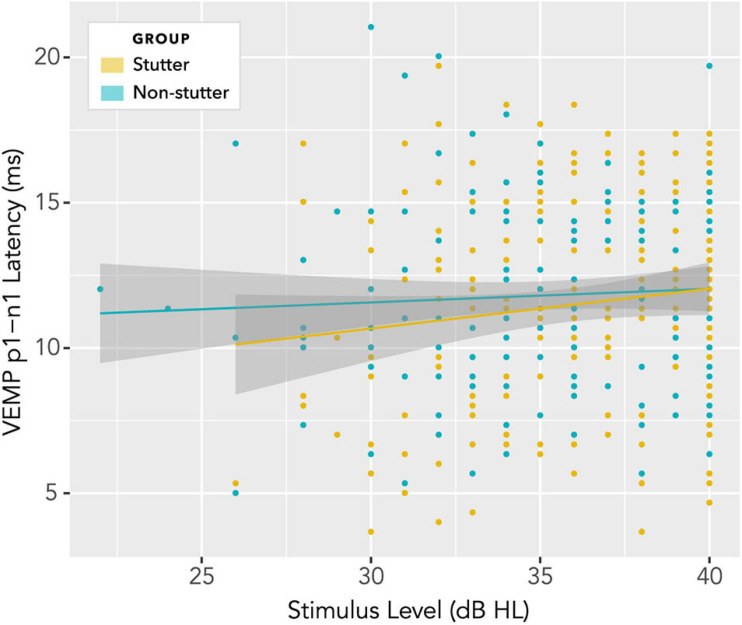
Variation of VEMP p1-n1 latency with stimulus level. There is no statistically significant interaction, and no indication of a group difference.

No statistically significant group differences were found for VEMP p1-n1 latency. Figure 12 shows latencies collected across all participants and all stimulus levels, including repeat measurements. Data appear normally distributed, with no indication of a group difference. Variation across participants with stimulus level is shown in Figure 13. There is no statistically significant interaction. Pearson’s correlation coefficient between VEMP p1-n1 latency and stimulus level is *r* (165) = 0.13, *p* = 0.10, 95% CI [−0.03, 0.27] for the stutter group, and *r*(165) = 0.06, *p* = 0.46, 95% CI [−0.10, 0.21] for the non-stutter group.

Group comparisons were evaluated through linear mixed-effects regression modelling, with *p*-values generated by likelihood ratio comparisons between the following models:

model_null: latency ∼ 1 + (1| participant)

model_diff: latency ∼ 1 + group + (1| participant)

There is no statistically significant difference between groups [chi squared (1) 0.07, *p* = 0.8].

This study had a pilot, described in more detail in the Supplementary Material. Similar analysis on pilot data shows no statistically significant difference between groups [chi squared (1) 2.6, *p* = 0.10].

## Discussion

Clinical presentation of stuttering is not accompanied by reports of difficulty with balance or dizziness (Bloodstein et al., 2021). As such, it is to be expected that clinical appraisal of the vestibular system in stutter and non-stutter groups should give broadly comparable results. This expectation is borne out in the box plots of Figures 7 and 8, and through the scaling of VEMP p1-n1 amplitude with stimulus level shown in Figure 10. On the basis of the current study, the vestibular clinician need make no particular allowance for stuttering when assessing clients who present with balance or dizziness complaints.

Nevertheless, there is a statistically significant finding that VEMP p1-n1 amplitude is 8.5 dB smaller in the stutter than the non-stutter group (*p* = 0.035, 95% CI [−0.9, −16.1], *t* = −2.1, *d* = −0.8). Whilst not of clinical importance for gravitoinertial function, the group difference will be interpreted in what follows according to its implications for speech-motor function in stuttering.

It will first be necessary to consider exactly what the group difference represents. The linear mixed-effects regression analysis compares two variables, both of which have been normalised relative to a background reference and transformed logarithmically (see “Data Processing”). It is the relationship between the transformed variables which is linear. Without the normalisation and transformation, the relationship between VEMP p1-n1 amplitude in volts and sound pressure in pascals would be described by a power law function. When viewed graphically, the logarithmic transformation will visually reduce differences between groups. The visual transformation can be difficult to interpret. This situation affects the box plots of Figures 7 and 8, and the linear plot of Figure 10. In all of these, a VEMP p1-n1 increase of 6 dB RL would correspond to a doubling of VEMP p1-n1 amplitude in microvolts (or more precisely, normalised microvolts – VEMPs are scaled per participant such that background is unity, as described in “Data processing,” so amplitudes are technically dimensionless ratios). When viewed without the logarithmic transformation, as in the VEMP wave form of Figure 4, the VEMP p1-n1 amplitude in the non-stutter group is twice as big as that in the stutter group.

As already remarked, a smaller VEMP p1-n1 amplitude in the stutter group than the non-stutter group need not be indicative of a difference in gravitoinertial function between stutter and non-stutter groups. Nevertheless, a smaller VEMP p1-n1 amplitude has implications for the way that own voice is perceived. With the logarithmic transformations in this report, an increment of 1 dB in stimulus level applied to the cochlea corresponds to a 2.1 dB increase in VEMP p1-n1 amplitude (see Figure 11 and the section “Electromyography”). Thus, the 8.5 dB group difference measured in VEMP p1-n1 amplitude means that stimulus levels for the stutter group need to be 4 dB higher than the non-stutter group (i.e., 8.5 ÷ 2.1) in order to produce an identically sized VEMP p1-n1 response. However, stimuli in this experiment were delivered through body conduction only. Stimuli during vocalisation contain an air conducted component of approximately equal magnitude to the body conducted component (Békésy, 1949; Reinfeldt et al., 2010). Thus, during vocalisation the stimulus level at the cochlea needs to be 8 dB higher in the stutter than the non-stutter group (i.e., 4 dB body conduction + 4 dB air conduction) in order to produce an identically sized VEMP p1-n1 response. When interpreting sound pressure level dB scales applied to the cochlea, a 10 dB increase corresponds to an approximate perceptual doubling (Stevens, 1972; Warren, 1973; Florentine et al., 2010). Given that spectral characteristics of the brief duration stimuli used in this investigation are within the human voice frequency range, the indication is that, for the stutter group, own voice perceived via the cochlea must be approximately twice as loud as for the non-stutter group in order to produce an identically sized VEMP p1-n1 response.

The remainder of this discussion will appraise three candidate explanations for the finding. The first two concern the possibility of the smaller VEMP p1-n1 response in the stutter group than the non-stutter group co-occuring with, or being a consequence of, differences between stutter and non-stutter groups in corticofugal activity or motor threshold subtentorially. The third possibility is that the smaller VEMP p1-n1 response in the stutter than the non-stutter group is indicative of a difference between stutter and non-stutter groups in an ascending neural stream corresponding to own voice, and that such a difference contributes to stuttering.

### Explanation 1: VEMP Response Modified by Differences in Corticofugal Activity Between Stutter and Non-Stutter Groups

Cortical research has indicated a motor threshold difference between stutter and non-stutter groups (Alm et al., 2013; Neef et al., 2015b; Busan et al., 2020). If a motor threshold difference between stutter and non-stutter groups affects brainstem reflexes, it might be possible to develop an explanation of why VEMP p1-n1 amplitude is smaller in the stutter group than the non-stutter group.

In the section “Background,” literature was summarised indicating that the VEMP should be considered as a short latency fragment of the vestibulo-collic reflex. A feature of this type of brainstem reflex (i.e., a reflex with no cortical involvement) is the rapidity of motor response compared to that which could be expected if cortical involvement was necessary. Functions such as balance and stability of gaze depend on such rapidity. Given that presentation of stuttering is not accompanied by reports of difficulty with gravitoinertial function, and that cerebral activity is not considered part of vestibular reflexes, the proposal that corticofugal activity affects VEMP response in people who stutter does not appear promising.

Nevertheless, corticofugal activity or the absence thereof can influence vestibular reflexes. McCall et al. (2017) review studies in which decerebration in animals, or strokes interrupting corticobulbar projections in humans, alter the gain of vestibulospinal reflexes and the response of neurons in vestibular nuclei. However, even in cases of chronic supratentorial stroke with spastic hypertonia unilaterally, asymmetry ratio in VEMP p1-n1 amplitude is one half or less between unaffected and affected sides (Miller et al., 2014). This is comparable to or less than the VEMP p1-n1 amplitude difference found between stutter and non-stutter groups in the current study (Figure 4). It moreover has the opposite direction of fit to that which might be expected. Alteration of corticofugal activity following a variety of supratentorial insults was found to increase VEMP p1-n1 amplitude, with the size of the increase corresponding to the amount of spasticity. Whereas in the stutter group for the current study, VEMP p1-n1 amplitude was decreased relative to the non-stutter group.

If differences in supratentorial structure or function between stutter and non-stutter groups contribute to differences in VEMP p1-n1 amplitude then, on the model of chronic stroke with spastic hypertonia, an increase in VEMP p1-n1 amplitude in the stutter group relative to the non-stutter group would be expected. Yet the opposite is found: VEMP p1-n1 amplitude is smaller in the stutter group than the non-stutter group.

For this reason, along with the aforementioned understanding (see section “Background”) that the vestibulo-collic reflex corresponds to activity in the vestibular brainstem and periphery, a cortical motor threshold difference between stutter and non-stutter groups does not appear workable as the basis for an explanation of group difference in VEMP p1-n1 amplitude. Following these considerations, an account of current findings which involves corticofugal activity seems unlikely to be compelling.

### Explanation 2: VEMP Response Modified by a Lower Subtentorial Motor Threshold in the Stutter Than the Non-stutter Group

An alternative explanation for the smaller VEMP response in the stutter group than the non-stutter group is that it is an artefact of a difference from the non-stutter group in motor threshold subtentorially. This would follow the suggestion of Zimmermann (1980) that a higher gain in brainstem reflexes contributes to stuttering.

Brainstem reflexes can be assessed through the startle response (Fetcho and McLean, 2009), a whole body flexor reaction to abrupt and intense stimulation. The startle response can be elicited by acoustic stimuli (e.g., bursts of white noise at 100 dBA) with measurement through the orbicularis oculi muscle which causes eye blink (Gómez-Nieto et al., 2020). When the startle stimulus is preceded by a smaller stimulus, referred to as a pre-pulse, the startle response is diminished. Experiments manipulating pre-pulse inhibition are used to appraise sensory gating (Cromwell and Atchley, 2015), a process in which stimuli are proposed to be filtered through ascending neural pathways such that cognitive processes will operate over a limited range of environmentally relevant percepts. Reduction in sensory gating would affect dopaminergic pathways and the striatum (Kaji et al., 2005), and may be accompanied by excessive attribution of salience to environmental stimuli. Such alterations to sensory gating may be present in neuropsychiatric diagnoses such as schizophrenia (Geyer, 2006). There may also be relevance to stuttering. Stuttering is thought to be accompanied by alterations in dopaminergic pathways (Alm, 2004; Alm and Risberg, 2007) and a difference between stutter and non-stutter groups in auditory sensory gating could potentially explain why altering audition during ongoing speech reduces the amount of stuttering (Cherry et al., 1956; Yates, 1963; Howell et al., 1987).

The startle response is modulated by the amygdala and stria terminalis (Davis et al., 1997) and can be altered by emotional context (Lang et al., 1990; Grillon and Baas, 2003). Alterations to the size of startle response can accompany post-traumatic stress disorder, mood and anxiety disorders, and traits related to anxiety and depression. However, the direction of change is not consistent (Vaidyanathan et al., 2009). Increased startle response is found in individuals having social anxiety (Pause et al., 2009). Several studies suggest increased anxiety in people who stutter (Craig, 1990; Craig et al., 2003; Ezrati-Vinacour and Levin, 2004) with overlap between the behavior of people who stutter and criteria for a diagnosis of social anxiety (Iverach et al., 2017). Although it is unclear whether anxiety in people who stutter is causative of stuttering, or is a result of the experience of stuttering, there is incentive to investigate acoustic startle response in participants who stutter.

For the reasons already described, acoustic startle has been compared several times between stutter and non-stutter groups. Guitar (2003) found a larger eye blink response in a stutter group than a non-stutter group, along with a higher score on the “nervous” subscale of the Taylor–Johnson Temperament Analysis (Taylor and Morrison, 1996). Pre-pulse inhibition was not tested. Alm (2006) and Alm and Risberg (2007) did not find a difference in eye blink response between stutter and non-stutter groups, including in tests of pre-pulse inhibition. Ellis et al. (2008) and Selman and Gregg (2020) also did not find a difference in acoustic startle between stutter and non-stutter groups. Alm and Risberg (2007) and Selman and Gregg (2020) also assessed temperament of participants using standardised instruments, and did not find group differences. On balance, the indication is that acoustic startle response does not differ between stutter and non-stutter groups.

A difficulty in assessing acoustic startle response in participants who stutter is that uncomfortable loudness levels have been found as lower in stutter groups than in non-stutter groups (MacCulloch and Eaton, 1971; Brown et al., 1975). In a study of non-stutter groups with and without tinnitus, acoustic startle response was found to increase as uncomfortable loudness level decreased (Knudson and Melcher, 2016). This was found in both tinnitus and non-tinnitus groups. The study also included anxiety and depression test batteries, finding no difference between groups and no correlation with either acoustic startle response or uncomfortable loudness level. Tests of acoustic startle response in participants who stutter have not evaluated uncomfortable loudness level, which will act as a confounder. Based on uncomfortable loudness level alone, an increase in acoustic startle response might be expected in stutter groups. However, such a finding would not necessarily inform understanding of anxiety, dopaminergic pathways or sensory gating in stuttering; it may simply be a side effect of a lower uncomfortable loudness level. In any event, increased acoustic startle response has only been found in one study involving a stutter group (Guitar, 2003), with four studies finding no group difference from a non-stutter group (Alm, 2006; Alm and Risberg, 2007; Ellis et al., 2008; Selman and Gregg, 2020).

In addition to the considerations already described, the vestibulo-collic reflex evaluated in the current study is not thought to have substantial overlap with the acoustic startle response. Firstly, the VEMP p1 latency of 10–15 ms is shorter than the 50 ms latency typical of the acoustic startle response (Bickford et al., 1964). Secondly, VEMPs can be driven at high rates of repetition (5.1 per second in the current study), unlike startle responses which, by definition, habituate rapidly (Landis and Hunt, 1939). A final point is that the 500 Hz body-conducted tone burst stimulus used in the current study had a maximum level of 40 dB HL. It thus contained energy well below the 100 dBA broadband stimuli used in acoustic startle studies, and would not be expected to generate a startle response.

In summary, there is not a compelling argument that the vestibulo-collic reflex evaluated in the current study is a component of the acoustic startle response, nor is there a convincing case that acoustic startle differs between stutter and non-stutter groups.

### Explanation 3: Corticopetal Activity in the Stutter Group Modified by a Smaller Vestibular Sensory Input During Vocalisation Than in the Non-stutter Group

Rather than a generally higher gain in brainstem reflexes, as considered in explanation two, subtentorial differences between the stutter and non-stutter groups may centre around own voice identification. Gattie et al. (in preparation) proposes that own voice is identified through coincidence detection between ascending neural streams of cochlear and vestibular origin. The proposal overlaps with explanation two, providing a basis for higher brainstem gain and reduced sensory gating. However, the proposal is restricted to own voice stimuli, and does not require involvement of the acoustic startle response.

From this perspective, subtle differences between stutter and non-stutter groups in auditory function would be side effects or neurodevelopmental consequences of a difference in own voice identification. At the brainstem or periphery these include auditory brainstem response (described later in this section), sound source localisation (Rousey et al., 1959), interaural phase disparity (Stromsta, 1972) and uncomfortable loudness levels (Brown et al., 1975). See Rosenfield and Jerger (1984) for further review. Literature describing how the amount of stuttering can be reduced with alterations to audition during ongoing speech is also germane (see Lincoln et al., 2006 or Foundas et al., 2013 for appraisal of clinical application, as well as citations in the introduction to this article). Differences between stutter and non-stutter groups are also found in auditory functions having cortical involvement. These include masking level (Liebetrau and Daly, 1981), backward masking (Howell et al., 2000; Lotfi et al., 2020) and dichotic listening tests (Sommers et al., 1975; Cimorell-Strong et al., 1983; Blood, 1985; Blood et al., 1987; Dmitrieva et al., 2000; Foundas et al., 2004). Blood oxygen level dependent tests of auditory function show differences in functional lateralisation between stutter and non-stutter groups (Sato et al., 2011; Halag-Milo et al., 2016). Electroencephalography and magnetoencephalography show differences between stutter and non-stutter groups in auditory oddball (P300; Morgan et al., 1997; Kaganovich et al., 2010; Jerônimo et al., 2020); auditory sensory gating (P1/P50m; Kikuchi et al., 2011); mismatch negativity (Corbera et al., 2005; Jansson-Verkasalo et al., 2014; Jerônimo et al., 2020); and alterations to timing and/or amplitude of the N1/M100 during listening tasks (Ismail et al., 2017; Kikuchi et al., 2017) and speech tasks (Salmelin et al., 1998; Beal et al., 2010, 2011; Liotti et al., 2010). Conflicting results are sometimes reported (e.g., Blood and Blood, 1984; Anderson et al., 1988; Khedr et al., 2000; Hampton and Weber-Fox, 2008; Özcan et al., 2009).

Other than the current study, there is only one investigation of the vestibular system in participants who stutter. Rotary chair testing showed no difference between stutter and non-stutter groups in a non-speech condition. However, during a speaking task, evoked horizontal nystagmus was found to be significantly more pronounced in a stutter group than a non-stutter group (Langová et al., 1975) exhibiting a pattern consistent with stellar nystagmus (Langová et al., 1983). Contemporary accounts in neuro-ophthalmology localise stellar nystagmus to the midbrain (Liu et al., 2018). Together with the current study, the suggestion is that during vocalisation there is a difference in the nature of subtentorial ascending activity, and/or conduction along the VIII cranial nerve, between stutter and non-stutter groups.

**FIGURE 14:**
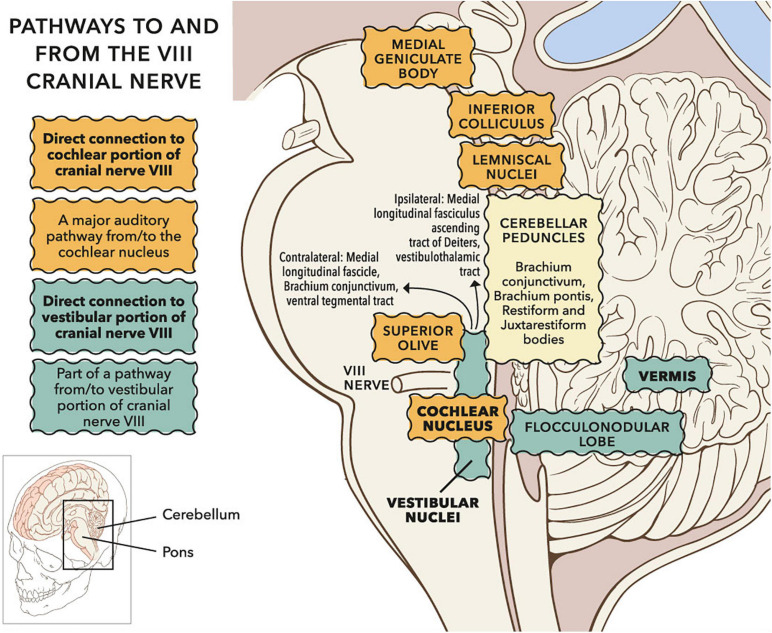
Sagittal view of subcortical pathways to and from the VIII cranial nerve. Whilst the auditory pathway ascending from the cochlear nucleus is relatively well established (Irvine, 1992), pathways to and from vestibular nuclei remain under investigation (Pierrot-Deseilligny and Tilikete, 2008; Zwergal et al., 2009). Projections to vestibular cortex via the thalamus have been investigated in humans through clinical observation and lesion studies (Conrad et al., 2014; Hitier et al., 2014; Wijesinghe et al., 2015). Vestibular nuclei also project down the spine (not shown). © Portions of this figure were adapted from illustrations by Patrick J. Lynch, http://patricklynch.net/. Creative Commons 2.5 license.

Figure 14 shows neural pathways connecting with the VIII cranial nerve in the brainstem and cerebellum. Vestibular fibres in the VIII cranial nerve predominantly terminate in vestibular nuclei. However, vestibular fibres also innervate cerebellar vermis, and sometimes flocculus (see review of amniotes in Newlands and Perachio, 2003). Govender et al. (2020) describe vestibular cerebellar evoked potentials in a non-stutter group using air- and body-conducted tone bursts (a stutter group was not tested). The evoked potentials have latencies between 10 and 20 ms and are likely to reflect climbing fibre responses via crossed otolith-cerebellar pathways. Climbing fibres enter the cerebellum through the inferior cerebellar peduncle, forming synapses with Purkinje cells. Vestibular nuclei are bidirectionally connected to the cerebellum, with investigation of pathways ongoing (Grüsser-Cornehls and Bäurle, 2001; Büttner-Ennever and Gerrits, 2004). Cerebellar vermis has repeatedly been identified as having differing activations in between participant comparisons of stutter and non-stutter groups during fluent speech, and in within participant comparisons of stutter groups during fluent and dysfluent episodes (Budde et al., 2014; Belyk et al., 2015).

Vestibular fibres also innervate the cochlear nucleus, either directly (Newlands and Perachio, 2003; Newlands et al., 2003) or via vestibular nuclei (Smith, 2012). The cochlear nucleus is the initial relay in a subcortical chain referred to as the ascending auditory pathway (Irvine, 1992). Electroencephalographic (EEG) activity in the ascending auditory pathway, following sound and vibration stimuli, is typically assessed through the auditory brainstem response (ABR). Stutter groups show greater differences in ABR from non-stutter groups when stimuli resemble speech (Tahaei et al., 2014; Crivellaro Goncalves et al., 2015; Mozaffarilegha et al., 2019) than when stimuli are clicks (Stager, 1990; Suchodoletz and Wolfram, 1996). However, all testing to date has been below clinical vestibular threshold, whereas clinical vestibular threshold will be exceeded during vocalisation (Todd et al., 2008; Curthoys et al., 2019). When sound stimuli are above vestibular threshold an additional component, N3, is present in the ABR (Mason et al., 1996; Nong et al., 2000, 2002; Papathanasiou et al., 2003, 2004, 2006; Murofushi et al., 2005). The nature of N3 has not been appraised in ABR tests of stutter groups.

Change in EEG morphology when stimuli exceed clinical vestibular threshold is seen cortically as well as in the auditory brainstem. When sound stimuli exceed clinical vestibular threshold, cortical EEG recordings show an additional component, the N42/P52, immediately prior to N1 (Todd et al., 2014b). The likely origin of N42/P52 is temporal or cingulate cortex (Todd et al., 2014a). As with the N3 in ABR, the nature of N42/P52 has not been investigated in stutter groups. However, the N1 has been important in investigations of stutter groups. The N1 (or its M100 equivalent in magnetoencephalography) is frequently used to evaluate speech-induced suppression (Houde and Nagarajan, 2016), in which temporal cortex activity during vocalisation is hypothesised to be moderated by speech-motor activity. Several authors have proposed that a difference in such moderation, or in auditory-motor mapping, between stutter and non-stutter groups underlies stuttering behavior (Max et al., 2004; Brown et al., 2005; Hickok et al., 2011; Cai et al., 2012). Such proposals have not been supported in direct tests evaluating N1/M100 amplitude (Beal et al., 2010, 2011; Liotti et al., 2010). However, all tests to date have used stimuli below clinical vestibular threshold. EEG morphology comparisons with stimuli above clinical vestibular threshold have not been made between stutter and non-stutter groups using either brainstem or cortical tests.

**FIGURE 15:**
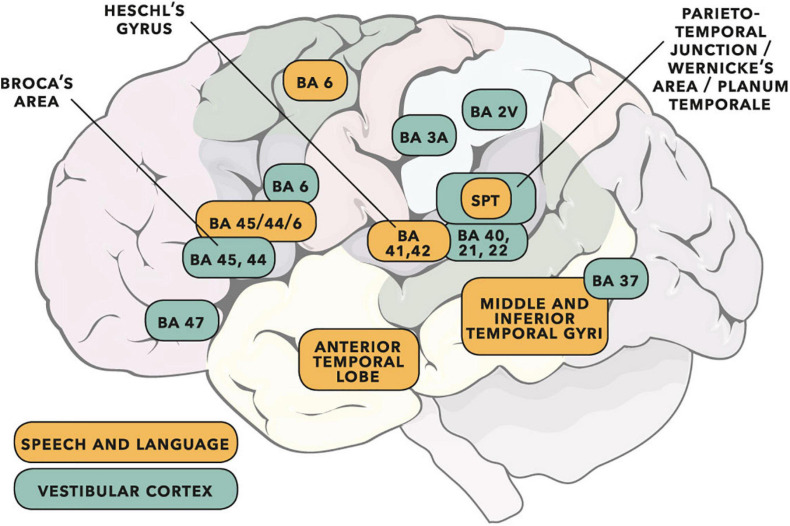
Cortical areas important for speech and language (adapted from the dual-stream model of Hickok and Poeppel, 2007) shown with vestibular cortical areas identified in cats, monkeys and humans (adapted from Ventre-Dominey, 2014; see also Frank and Greenlee, 2018). Cortical activity following vestibular input has wide interpretation (e.g., see reviews of cognition in Hitier et al., 2014, and audition/rhythm/timing in Todd and Lee, 2015). Some of the vestibular areas identified will be predominantly related to gravitoinertial function (see discussion in Ferrè and Haggard, 2020). Numbers are Brodmann areas – see primary literature for more exact location detail. Spt is the Sylvian temporo-parietal region proposed by Hickok and Poeppel (2007) as a sensorimotor integration area. Vestibular sites in humans have been identified as such when direct electrical stimulation of the cortex gives rise to gravitoinertial illusion. When vestibular sites are identified within BA 21 (lateral temporal lobe) or BA 22 (Wernicke’s area), auditory illusion is found to accompany gravitoinertial illusion (Kahane et al., 2003; Fenoy et al., 2006). © Portions of this illustration were adapted from Servier Medical Art, https://smart.servier.com. Creative Commons 3.0 license.

Figure 15 overlays cortical areas identified through study of the vestibular system and cortical areas found to be important for speech and language. Overlap is apparent in several areas. Based on the literature reviewed in this section, there is substantial motivation for a more detailed appraisal of the vestibular system in participants who stutter.

### Other Diagnoses in Which VEMP Tests Show a Difference From Control Participants

VEMPs are typically used as part of a diagnostic test battery following balance and dizziness complaints (Rosengren et al., 2019). A difference from controls in VEMP testing can additionally be used to support diagnoses which perhaps have no obvious relation to balance and dizziness, or to each other. These include brainstem lesions (Oh et al., 2013), multiple scleroris (Escorihuela García et al., 2013; Gabelić et al., 2013; Ivanković et al., 2013; Güven et al., 2014), dementia (Harun et al., 2016), Parkinson’s disease (Shalash et al., 2017) and attention deficit and hyperactivity disorder (Isaac et al., 2017). See Oh et al. (2016) or Deriu et al. (2019) for further review and discussion. Gattie et al. (in preparation) discusses how brain areas identified as having structural or functional importance in participants who stutter may be common with brain areas identified in diagnoses which have a higher than chance overlap with stuttering.

### Limitations of the Current Study

This report would benefit from replication with a higher participant count. However, the statistical analysis is more compelling than might typically be the case for a pre-registered case control study of this size (15 stutter, 15 non-stutter). For example, if two participants with a stuttering presentation and/or history differing from others in the stutter group had not been included in the analysis, a larger group difference of 11.2 dB (*p* = 0.007, 95% CI [−3.6, −18.9]) would have been reported. Furthermore, 7 of the 15 controls were representative of a normative sample of 48; and the pilot study (five participants who stutter, five non-stutter controls) had near-identical results to the main study (10.1 dB group difference, *p* = 0.044, 95% CI [−1.3, −18.9]). The finding of a difference in vestibular function between stutter and non-stutter groups is in agreement with the only prior research on the vestibular system with participants who stutter (Langová et al., 1975).

## Conclusion

Vestibular-evoked myogenic potential was found to have a significantly smaller p1-n1 amplitude in a stutter group than a non-stutter group. Although not of clinical importance with regard to gravitoinertial function, the group difference may have importance for understanding of speech-motor function in participants who stutter. The finding of a difference in vestibular function between a stutter and a non-stutter group is consistent with prior research on the vestibular system in stuttering (Langová et al., 1975). Review of vestibular pathways, and in particular the response of the vestibular system to sound and vibration, motivates further investigation of the vestibular system in participants who stutter. There is overlap between brain areas receiving vestibular innervation, and brain areas identified as important in studies of stuttering. These include the auditory brainstem, the cerebellum and the temporo-parietal junction.

This study was pre-registered as predicting a difference in VEMP between stutter and non-stutter groups. The pre-registration gives the disruptive rhythm hypothesis (Howell et al., 1983; Howell, 2004) as a rationale. The disruptive rhythm hypothesis proposes that sensory inputs additional to own speech audition will be maximally fluency-enhancing when they coordinate with ongoing speech. The disruptive rhythm hypothesis is supported by this study. Vestibular input which coordinates with ongoing speech is fluency enhancing in ordinarily fluent controls, whereas the smaller vestibular input in people who stutter results in less fluency enhancement, accounting for the observed stuttering behavior.

The study was motivated by a hypothesis which is compatible with, and adds detail to, the disruptive rhythm hypothesis (Gattie et al., in preparation). The basis of the hypothesis is that coincidence detection between deflection of cochlear and vestibular mechanoreceptors during vocalisation is fundamental to own voice identification, and that own voice identification differs between stutter and non-stutter groups.

## Data Availability Statement

The raw data supporting the conclusions of this article will be made available by the authors, without undue reservation.

## Ethics Statement

The studies involving human participants were reviewed and approved by the University of Manchester Ethics Committee. Written informed consent to participate in this study was provided by the participants. All participants were adults. They provided written informed consent themselves, rather than through guardians or next of kin.

## Author Contributions

MG: conceptualisation (ideas and formulation of the overarching research goals and aims), software (programming, software development, designing computer programs, implementation of computer code or algorithms, and testing code components), investigation (conducting the research and investigation process, specifically performing the experiments, or data collection), data curation [annotation, scrubbing, or maintenance of research data (including software code, where it is necessary for interpreting the data itself)], writing—original draft (preparation, creation and/or presentation of the published work, specifically writing the initial draft), visualisation (preparation, creation and/or presentation of the published work, specifically data presentation or visualisation). MG, KK, and EL: methodology (development or design of methodology or creation of models), validation (verification of the replication and reproducibility of results, experiments, or other research outputs), formal analysis (application of statistical, mathematical, computational, or other techniques to analyse, or synthesise data), resources (provision of study materials, materials, instrumentation, computing resources, or analysis tools), writing—review and editing (critical review, commentary, or revision), project administration (coordination of the research activity planning and execution), and funding acquisition (acquisition of financial support). KK and EL: supervision (oversight and leadership responsibilities and including mentorship). All authors contributed to the article and approved the submitted version.

## Conflict of Interest

The authors declare that the research was conducted in the absence of any commercial or financial relationships that could be construed as a potential conflict of interest.

## Publisher’s Note

All claims expressed in this article are solely those of the authors and do not necessarily represent those of their affiliated organizations, or those of the publisher, the editors and the reviewers. Any product that may be evaluated in this article, or claim that may be made by its manufacturer, is not guaranteed or endorsed by the publisher.
