# Supplementary material for: Weak Vestibular Response in Persistent Developmental Stuttering
Source: Front Integr Neurosci. 2021 Sep 1;15:662127. doi: 10.3389/fnint.2021.662127 (PMC8477904; doi:10.3389/fnint.2021.662127)
Supplement: Supplementary file 1 [file Data_Sheet_1.pdf]

Supplementary Material for:
Weak vestibular response in persistent developmental stuttering

Corresponding Author: max.gattie@manchester.ac.uk

Contents

1. Participant Details
2. Pre-stimulus Electromyographic Root Mean Square
3. Alternative Linear Mixed Model analyses of VEMP p1-n1 amplitude
4. Pilot Study

1 Participant Details

For all participants, the following tests were conducted prior to electrophysiological data collection:

- Otoscopic examination of the tympanic membrane.
- Middle ear impedance at 226 Hz, using an Interacoustics Titan IMP440 (Interacoustics, Denmark).
- Pure tone audiometry over air- and bone-conduction (AC/BC) using a GSI-3B Arrow™ audiometer (Grason-Stadler Inc., MN, USA) with TDH49 headphones (Telephonics Corp., Farmingdale, NY, USA) or a B71 bone conductor (Radioear, MN, USA). Participants were evaluated for thresholds below 20 dB nHL at 1000 Hz, 500 Hz, 250 Hz and 125 Hz (AC), and 1000 Hz, 500 Hz and 250 Hz (BC).

Testing followed British Society of Audiology recommendations. Three participants were excluded because deposits of cerumen were sufficiently substantial as to occlude view of the tympanic membrane. For one of these participants, tympanometry could not be performed successfully. All other participants presented with normal hearing according to the tests described. One participant was excluded due to a growth on the mastoid bone which prevented use of the bone conductor. Replacements were recruited for excluded participants.

Participants were recruited differently for the two experimental groups.

Participants who stutter (PWS) were recruited from the Manchester Stammering Support Group. PWS self-referred to the group, either following a suggestion to do so from British National Health Service centres where they had received therapy for stuttering, or following discovery of the group via internet search. All PWS identified as stuttering upon presentation at support group meetings, and were only invited to participate after the lead author had observed them displaying overt stuttering behaviour. Unimpaired hearing was confirmed via self-report before the invitation to participate. PWS were compensated for participating in the two hour session in line with 2019 UK Living Wage Foundation recommendations.

Ordinarily fluent control speakers were recruited from two groups. The 7 participants aged less than 21 years old were drawn from a normative data set of 48 psychology undergraduates at the University of Manchester, who participated in return for course credit. As well as exact pairing on sex, and near-exact pairing on age (see table 3) the availability of 48 control participants created an opportunity for selection of controls based on an additional criterion. Since VEMP p1-n1 amplitude is the measure of interest in the comparison of people who do and do not stutter, controls were chosen such that their average VEMP p1-n1 amplitude would be representative of that for the normative data set. There is no significant group difference for VEMP p1-n1 amplitude based on sex (24 female, 24 male) in the normative data set (Gattie, Lieven & Kluk, in preparation). Therefore the 7 control participants were selected to have VEMP p1-n1 amplitudes representative of the entire normative data set of 48, rather than being a normative sample of 7 as would have been the case if controls aged younger than 21 years had been sampled randomly from the general population. Selection based on VEMP p1-n1 amplitude was carried out simultaneous with pairing on sex and age, with the 7 controls chosen such that their averaged VEMP p1-n1 amplitude across all stimulus levels tested (mean 48.8 dB RL, SD 14.8) was comparable to that of the 40 participants not chosen from the normative sample (mean 48.7 dB RL, SD 14.2). Participants older than 21 years were postgraduates at the University of Manchester, and participated without compensation.

Control participants were told that the experiment was about stuttering, and asked if they stuttered. No control participants identified as stuttering (if they had done, availability of the stuttering support group would have been explained to them). In all cases, unimpaired hearing was confirmed via self-report before the invitation to participate.

Stuttering was appraised quantitatively using the SSI-4 (Riley, 2009). The SSI-4 is a norm-referenced stuttering measure based around count of stuttered syllables. Criteria were that controls would be excluded with an SSI-4 score above 10, and PWS would be excluded with an SSI-4 score below 10. SSI-4 categorisations for controls and PWS are shown in table 1.

Group	SSI-4 score and rating					
	Do not stutter (<10)	Very mild (10 – 17)	Mild (18 – 24)	Moderate (25 – 31)	Severe (32 – 36)	Very severe (37 – 46)
Controls	15	–	–	–	–	–
PWS	–	–	6	4	3	2

Table 1: SSI-4 scores for ordinarily fluent controls and participants who stutter.

A self-assessment questionnaire (form 1, attached to this appendix) was used to assess whether anything other than stuttering might affect the results of the VEMP test. Any positive answers to the questionnaire were followed up at interview, along with additional probe questions (see interview sheet attached as form 2). The questionnaire and interview were not intended to provide data for quantitative analysis. The intention was partly as an additional screen (given that time constraints meant that all factors of potential interest could not be assessed via test batteries) and primarily to gather hypothesis-generating data. Assessment of the auditory brainstem response to clicks has suggested PWS may separate into one group within, and another without, the response range of matched controls (Stager, 1990). If VEMP responses had suggested a subgrouping of PWS similar to that for PWS auditory brainstem response to clicks, data collected via questionnaire and interview might have helped to design a follow-up study which could assess heterogeneity in PWS.

The results of the study (figure 6 box plot) showed only two cases where controls had clearly smaller VEMP p1-n1 amplitudes than a paired PWS. Whereas in 10 cases, VEMP p1-n1 amplitudes of PWS are overall markedly smaller than in paired controls. In the three remaining cases there was a partial overlap which, in the linear mixed models analysis, was part of a statistically significant group difference between PWS and paired controls for VEMP p1-n1 amplitude. There was therefore little suggestion that PWS should be sorted into subgroups based on VEMP response. As such, data collected from the questionnaire and follow-up interview will not be reported in full. Inferences from such data would be statistically invalid based on the small sample size in this study.

What can be reported, based on the questionnaire and interview, is that no participants reported or presented with non-speech conditions which could have affected test results. Education was at level 3 (British A-level or equivalent) or higher. Some individual reports from PWS included in the study are of interest:

- One PWS had a spinal condition which might have affected ability to complete the head bar push in the VEMP procedure. The participant had no difficulty completing the procedure, and VEMP recordings were similar to other PWS (i.e. VEMP p1-n1 amplitudes smaller than the paired control).
- Another PWS presented with cluttering in addition to stuttering. The SSI-4 indicated a positive diagnosis for stuttering, as did self-report of the PWS and the assessment of the lead author. The SSI-4 score was the lowest of any PWS tested (19, on a scale from 0 to 46 where scores greater than 10 are classified as stuttering), and VEMP p1-n1 amplitudes were higher than those of the paired control. This is perhaps not a typical case of persistent developmental stuttering (PDS). If this participant and the paired control had been excluded from analysis (i.e. on the basis that it is not a typical case of PDS), statistical analysis for the remaining 14 PWS with paired controls would have shown stuttering with an effect of -9.4 dB and $p = 0.020$ (compared to -8.5 dB and $p = 0.035$ using 15 PWS with paired controls).
- Another PWS presented with a history consistent with psychogenic stuttering (van Borsel, 2014). Psychogenic stuttering is a type of acquired stuttering, is

rarer than PDS, and is typically reported through case study (Baumgartner & Duffy, 1997). Age of onset was 10 years for this participant, which is relatively late for childhood stuttering (Bloodstein et al., 2021). Although acquired stuttering is typically considered an adult condition, there is no principled basis by which children should be considered unsusceptible to acquired stuttering (Theys et al., 2009). Thus, it is not clear that this participant had PDS. Despite this, the participant was included in the PDS group. The reason for this was that differential diagnosis is not straightforward (Baumgartner & Duffy, 1997; Ward, 2010), and was further hindered by the 18 year gap between stuttering onset and participation in the current study. The SSI-4 test indicated mild stuttering, and onset at 10 years old suggests PDS, so PDS is a fair categorisation. However, it is notable that this PWS was unusual in having a clearly larger VEMP p1-n1 amplitude than the paired control. If this PWS and paired control had been excluded from analysis (i.e. on the basis of not being a case of PDS), statistical analysis for the remaining 14 PWS with paired controls would have shown that stuttering has an effect of -10.1 dB and $p = 0.015$ (compared to -8.5 dB and $p = 0.035$ using 15 PWS with paired controls).

To summarise: if 2 of the 15 PWS (with paired controls) had been excluded on the basis that their stuttering was atypical of PDS, statistical analysis for the remaining 13 PWS with 13 paired controls would have shown stuttering with an effect of -11.2 dB ($p = 0.007$, 95% CI -3.6, -18.9), compared to -8.5 dB ($p = 0.035$, 95% CI -0.9 to -16.1) with 15 PWS and 15 paired controls.

Full details of pairing are shown in table 2. For cervical VEMPs, p1-n1 amplitude (in μV) in response to BC tone bursts falls linearly at 14.3% per decade once participants are older than 20 years (Colebatch et al., 2013). With the dB RL conversion in this report, the decline over a decade is 20 times the base 10 logarithm of (1 - 0.143), equating to 0.134 dB RL per year. PWS and controls were paired such that mean age difference was -0.1 years (SD 1.8). The mean mismatch in p1-n1 amplitude due to age difference between pairs is 0.01 dB RL. Age effects were thus considered to cancel in the between group comparisons, and age was not a factor in statistical analysis.

Participant ID	Sex	SSI-4	Group	Age (yrs)	difference (yrs)	dB RL mismatch
FZNN6543	Male	34	PWS	17.3		
CAMC0468	Male	8	CONT	18.8	1.4	-0.18
JOBA2769	Male	36	PWS	18.8		
JASP1911	Male	9	CONT	19.0	0.3	-0.04
NAHT5214	Male	19	PWS	19.8		
MAIT7329	Male	5	CONT	20.0	0.2	0.01
BAIB6567	Male	25	PWS	19.6		
JOPS4312	Male	4	CONT	19.6	0.0	0.00
IS4C4545	Male	38	PWS	20.9		
SCHW7222	Male	2	CONT	21.3	0.3	-0.04
SOO19192	Female	24	PWS	20.6		
JI7634	Female	4	CONT	20.2	-0.4	0.06
SAFA1218	Male	26	PWS	20.7		
NI6743	Male	2	CONT	20.8	0.1	-0.01
MILG4836	Male	39	PWS	22.5		
ISAB1291	Male	2	CONT	21.3	-1.2	0.16
HARR1598	Male	27	PWS	24.7		
LWIG0921	Male	0	CONT	23.7	-1.0	0.13
NIYT6794	Male	22	PWS	25.3		
HE8692	Male	6	CONT	26.2	0.9	-0.12
PA5679	Male	21	PWS	26.5		
RO8893	Male	0	CONT	26.1	-0.4	0.06
STEL5152	Female	27	PWS	26.0		
JE8779	Female	6	CONT	27.5	1.5	-0.20
GRTT6591	Male	23	PWS	28.3		
ADAD2376	Male	3	CONT	27.1	-1.3	0.17
SQRF5874	Male	35	PWS	36.5		
IB54347	Male	7	CONT	31.2	-5.3	0.52
JMJM5454	Male	21	PWS	37.0		
PEDN5219	Male	1	CONT	40.0	3.0	-0.40
mean (of positive/negative values)				24.2	-0.1 (SD 1.8)	0.01
mean (of absolute values)					1.2 (SD 1.4)	0.04

Table 2: PWS shown with paired controls (CONT). The estimated error in VEMP p1-n1 amplitude, due to age difference between pairs, is provided in dB RL.

STUTTERING STUDY
QUESTIONNAIRE

MANCHESTER 1824
The University of Manchester

PARTICIPANT NUMBER

It will help us if you can answer these questions as accurately as possible.

Sometimes you may be in doubt as to whether to reply yes or no. In these cases, please answer yes, and we will discuss your answer in a follow-up interview.

You do not have to answer any questions you do not want to answer. If you don't want to answer a question, leave it blank.

1. Do you tend to favour your right hand, your left hand, or neither hand?

   Right hand
   Left hand
   No hand preference

2. Have you noticed anything unusual about your hearing?

   Yes
   No

   If yes, please describe what's unusual:

3. Do you stutter, either now or in the past?

   Yes
   No

   If yes, please describe your history of stuttering:

4. Do you make any unusual or unexpected movements (e.g. tics)?

   Yes
   No

   If yes, please describe the movements:

5. How would you describe your sense of rhythm and coordination in comparison to your friends' sense of rhythm and coordination?

   Excellent
   Above average
   Average
   A little below average
   I have a rhythm of my own

   Please describe your sense of rhythm and coordination, if it is unusual:

PTO

IRAS Project ID 210702 1 VERSION 2 8 NOVEMBER 2016

Form 1: Self-assessment questionnaire, page 1.

6. Have you ever been diagnosed with any of the following disorders?

Articulation
Phonology
Voice
Cluttering
Stuttering
Dysphagia
Receptive language
Expressive language
Specific language impairment
Learning
Literacy
Attention deficit
Central auditory processing
Neuropsychological
Behavioural
Sensory integration
Acquired physical conditions
(e.g. diabetes or asthma)
Autistic spectrum
Epilepsy or other neurological disorder
Mental retardation
Hearing impairment
Congenital physical conditions
(e.g. cleft lip or palate)
Traumatic brain injury
Hormonal imbalance
Emotional disorder
Tourette's syndrome
Cerebral palsy

If you replied yes to any of the above, please give details:

7. Do you have any other medical history you think we should know about?

Yes
No

If yes, please describe the history:

8. Which of the following best describes your formal education?

Level 1 (e.g. GCSE grades D–G)
Level 2 (e.g. GCSE grades A*–C)
Level 3 (e.g. A level or AS level)
Level 4 (e.g. HNC)
Level 5 (e.g. HND)
Level 6 (e.g. Bachelor's undergraduate degree)
Level 7 (e.g. Master's degree)
Level 8 (e.g PhD)

Please return this form to:

Max Gattie
School of Psychological Sciences
Ellen Wilkinson Building
University of Manchester
Oxford Road, Manchester
M13 9PL

Tel: 0161 275 8567
email: max.gattie@postgrad.manchester.ac.uk

IRAS Project ID 210702 2 VERSION 2 8 NOVEMBER 2016

Form 2: Self-assessment questionnaire, page 2.

Participant Details

Unique identifier:

Name:

Date of birth:

Contact details:

Mother's education:

Languages:

Music experience:

Hearing history:

Recent loud noises:

Stuttering history:

Other details:

Head size:

- Maximum cross-sectional diameter
- Sagittal nasion to inion
- Coronal top section (from where pinna joins skull)

TURN YOUR PHONE OFF!

Form 3: Interview prompt sheet.

2 Pre-stimulus Electromyographic Root Mean Square

Although neck tension was tightly controlled throughout by use of the padded bar with biofeedback (see section 2.4) pre-stimulus neck tension was analysed to ensure that it had not acted as a confounder. This was achieved through the electromyographic (EMG) recording made with the Eclipse. Averaging of the 18 ms pre-stimulus interval per participant (see section 2.5) gave a mean pre-stimulus root mean square EMG amplitude of 2.7 µV (SD 1.1) for the stutter group and 3.1 µV (SD 1.3) for the non-stutter group. Note that these means are derived from 1800 presentations per participant, and are therefore much lower than the 50 µV biofeedback target that participants were asked to maintain according to the real time calculations in the Eclipse clinical software. The differences in pre-stimulus root mean square EMG amplitude can be used to calculate an upper limit for difference between stutter and non-stutter groups corresponding to pre-stimulus neck tension. This upper limit is 20 times the base 10 logarithm of (2.7 µV ÷ 3.1 µV), or -1.2 dB RL, relative to a group difference in VEMP p1-n1 amplitude of 8.5 dB RL. However, VEMP amplitude measurements have already been adjusted for variation in pre-stimulus root mean square EMG amplitude on a per participant basis through the normalisation routine (see section 2.5). As such, per participant differences in pre-stimulus root mean square EMG amplitude are already incorporated into the statistical analysis. No adjustment is necessary to the finding that VEMP p1-n1 amplitude is 8.5 dB smaller in the stutter group than the non-stutter group ($p = 0.035$, 95% CI [-0.9, -16.1], Chi-Squared (1) = 4.44, $d = -0.8$, conditional $R^2 = 0.88$). Indeed, any such adjustment would invalidate the statistical analysis. If there was a worry that pre-stimulus neck tension was acting as a confounder, the correct analysis is to include pre-stimulus root mean square EMG amplitude as a covariate (i.e. as in analysis of the pilot study described in section 4 of this supplementary material, in which pre-stimulus root mean square EMG amplitude was more variable than in the main study because neck tension was created via head raise from supine). For the main study, any worry over variation in pre-stimulus neck tension is removed by use of the head bar with biofeedback, and by the normalisation procedure. The group comparison of pre-stimulus root mean square EMG amplitude for the main study as reported here is purely to illustrate and emphasise that it could not have acted as a confounder.

At the suggestion of a reviewer, the pre-stimulus root mean square EMG amplitude for the two PWS whose stuttering was atypical of persistent developmental stuttering was checked to ensure that these participants had not been pushing harder than other participants on the head bar. In one of the participants, the pre-stimulus root mean square EMG amplitude was 2.6 µV (SD 1.4), which is almost identical to the mean of 2.7 µV (SD 1.1) for the stutter group as a whole. Thus, an atypical pre-stimulus root mean square EMG amplitude cannot explain the atypical result for this participant. In the other participant, the pre-stimulus root mean square EMG amplitude was 3.5 µV (SD 1.05). This is 0.8 µV higher than the mean of 2.7 µV (SD 1.1) for the stutter group, and is also higher than the 3.1 µV (SD 1.3) mean for the non-stutter group. However, the 0.8 µV difference relative to the 2.7 µV mean for the stutter group equates to an increase of 20 times the base 10 logarithm of (3.5 µV ÷ 2.7 µV), or 2.3 dB RL. This is insufficient to account for the difference in VEMP p1-n1 amplitude between the PWS and paired control, which was approximately 8 dB RL in favour of the PWS whereas -8.5 dB RL would have been consistent with the statistical analysis for the study. Thus, an atypical pre-stimulus RMS cannot explain the atypical result for this participant.

3 Alternative Linear Mixed Model analyses of VEMP p1-n1 amplitude

Visual inspection of figure 8 (Stimulus level versus VEMP p1-n1 amplitude per participant) shows broadly similar slopes. But perhaps some PWS have slopes differing from those of controls and of other PWS? The mean slope for the PWS group is 2.3 (SD 0.5), compared to 2.1 (SD 0.3) for controls. A two-sided t-test shows this difference is not statistically significant ($t(21) = 1.1$, $p = 0.30$) but a larger sample might indicate, for example, that the slope is constant in controls but differs in some PWS. If so, a varying intercept and slope model would be appropriate. The slope difference may convey physiological detail (e.g. that activity in vestibular mechanoreceptors or VIII cranial nerve scales differently between PWS and controls as stimulus level increases).

Linear mixed-effects models with fixed and varying slopes are summarised in table 3.

In these, *p* values were generated by likelihood ratio comparisons to a nested model in which the "isPWS" predictor (true if a participant stutters) was absent. All models were fitted using the "maximum likelihood" setting in lme4.

Linear Mixed Model	Include Outliers	Comments	**PWS-CONT diff** (std err)	**p value** [(df) χ^2]
**Fixed slope** logp1n1 ~ dB + isPWS + (1|PiD)	Yes		**-8.47** (3.87)	**0.035*** [(1) 4.44]
	No		**-8.14** (3.90)	**0.043*** [(1) 4.08]
**Varying slope** logp1n1 ~ dB + isPWS + (1 + dB|PiD)	Yes	Convergence warning	**-7.90** (3.72)	**0.049*** [(1) 3.87]
	No		**-8.74** (3.61)	**0.021*** [(1) 5.30]

Table 3: Linear mixed-effects regression modelling comparisons. All models have varying intercepts. Model description is as per the lme4 syntax: logp1n1 is the VEMP p1-n1 amplitude in dB RL; dB is the stimulus level in dB HL; isPWS is a binary indicator of PWS (true) versus control (false); PiD is the participant ID.

The simplest varying slope model creates a convergence warning. The warning can be overcome by removing 12 outlying data points (from 334 data points total; outliers are data outside the interquartile range, and are visible mostly towards the bottom of the figure 6 box plot). With outliers removed, there is no convergence warning, and PWS have a VEMP amplitude significantly smaller than paired controls by 8.7 dB (Chi-Squared (1) = 5.30, $p = 0.021$). Whichever model is chosen, the *p* value is below 0.05.

4 Pilot Study

4.1 Limitations compared to the main study

In the pilot for the main study, analysis of VEMPs used a clinical threshold estimation procedure (BSA, 2012) which established no statistically significant group difference between PWS and controls. Here, pilot data is reassessed using the linear mixed-effects regression analysis scripts developed for the main study.

Limitations of pilot study procedures, in comparison to those in the main study, are:

- Presentation was descending clinical threshold search (BSA, 2012), rather than counterbalanced even and odd descending sequences as in the main study.
- An earlier version of the EP25 software was used. This identified the Radioear B81 as the earlier model B71 bone conductor. The B81 is capable of an output between 11–16 dB higher than the B71 at 500 Hz (Jansson et al., 2015) but the earlier version EP25 software would not drive the Radioear B81 at such levels.
    - Stimulus levels for the pilot study should be considered as dial settings, since calibrations were manipulated to obtain the highest possible output from the B81.
    - The maximum stimulus level used in the pilot study is lower than that for the main study.
- SCM tension was generated via head raise from supine, and will be more variable than when using the head bar and biofeedback.

4.2 Participants

Participant recruitment for the pilot study was similar to that described in the main study, except that pairing on age and sex is only precise in aggregate (table 4). SSI-4 scores are shown in table 5.

Participant ID	Sex	SSI-4	Group	Age (yrs)	difference (yrs)	dB RL mismatch
IDAM2209	Female	33	PWS	24.4		
REET0304	Male	6	CONT	18.8	-5.6	-0.75
SKOT9050	Male	19	PWS	25.6		
FIZZ2001	Male	6	CONT	23.2	-2.4	-0.32
AERO7835	Male	25	PWS	35.2		
PHON8215	Male	3	CONT	36.7	1.5	0.20
JAGG5728	Male	18	PWS	37.6		
TDRR3645	Female	5	CONT	37.0	-0.6	-0.08
TVOY8855	Male	28	PWS	39.2		
BEAG1273	Male	2	CONT	46.7	7.5	1.01
mean (of positive/negative values)				32.4	-0.1 (SD 4.9)	0.01
mean (of absolute values)					3.5 (SD 2.9)	0.47

Table 4: Pilot study, PWS shown with paired controls. The estimated error in VEMP p1-n1 amplitude, due to age difference between pairs, is provided in dB RL.

Group	SSI-4 score and rating					
	Do not stutter (<10)	Very mild (10 – 17)	Mild (18 – 24)	Moderate (25 – 31)	Severe (32 – 36)	Very severe (37 – 46)
Controls	5	–	–	–	–	–
PWS	–	–	4	1	–	–

Table 5: Pilot study, SSI-4 scores for ordinarily fluent controls and participants who stutter.

Data collection for the pilot study featured 8 PWS and 8 controls, but three pairs were removed following inability to record a VEMP from one of the pair. Participants in these pairs had ages between 39 and 57 years. Inability to record VEMPs from these participants is likely to be due to VEMP amplitudes declining with age (Colebatch et al., 2013) and the smaller stimulus levels used in the pilot versus the main study (VEMP p1-n1 amplitudes are proportional to stimulus level; Todd et al., 2008; Dennis et al., 2016).

Two PWS (ages 24.4 and 25.6 in figure 14) from the pilot study were retested, with different controls, for the main study. Direct comparison between the pilot test and the main study in not possible due to differences in equipment calibration. In the pilot study, VEMP amplitudes for the two PWS were similar to those of matched controls, whereas when the two PWS were retested for the main study (ages 26.0 and 26.5 in figure 3) VEMP amplitudes were clearly lower than those of their pairs. However, variation due background neck tension is accounted for in the linear mixed-effects regression model of the pilot study (figure 17), but is not accounted for the box plot of figure 15. Thus, comparison of box plots between the main study and pilot study will mislead. Statistical analysis using linear mixed-effects regression models (figures 6 and 17) provides the most accurate assessment of the retested participants.

4.3 Results

Figure 16 suggests a normal distribution for VEMP amplitudes (given small sample size), and figure 17 shows box plots with participant matching.

Because the head bar was not used to control SCM tension, a model fit was chosen in which the pre-stimulus root mean square of EMG amplitude is used as an additional predictor corresponding to background SCM tension. This is consistent with the model described in figure 5. Application of the same random intercepts model used for the main study gives the statistically significant result that PWS have a VEMP amplitude 10.1 dB smaller than matched controls for the range of stimulus levels tested (p = 0.044, 95% CI [-1.3, -18.1], Chi-Squared (1) = 4.05).

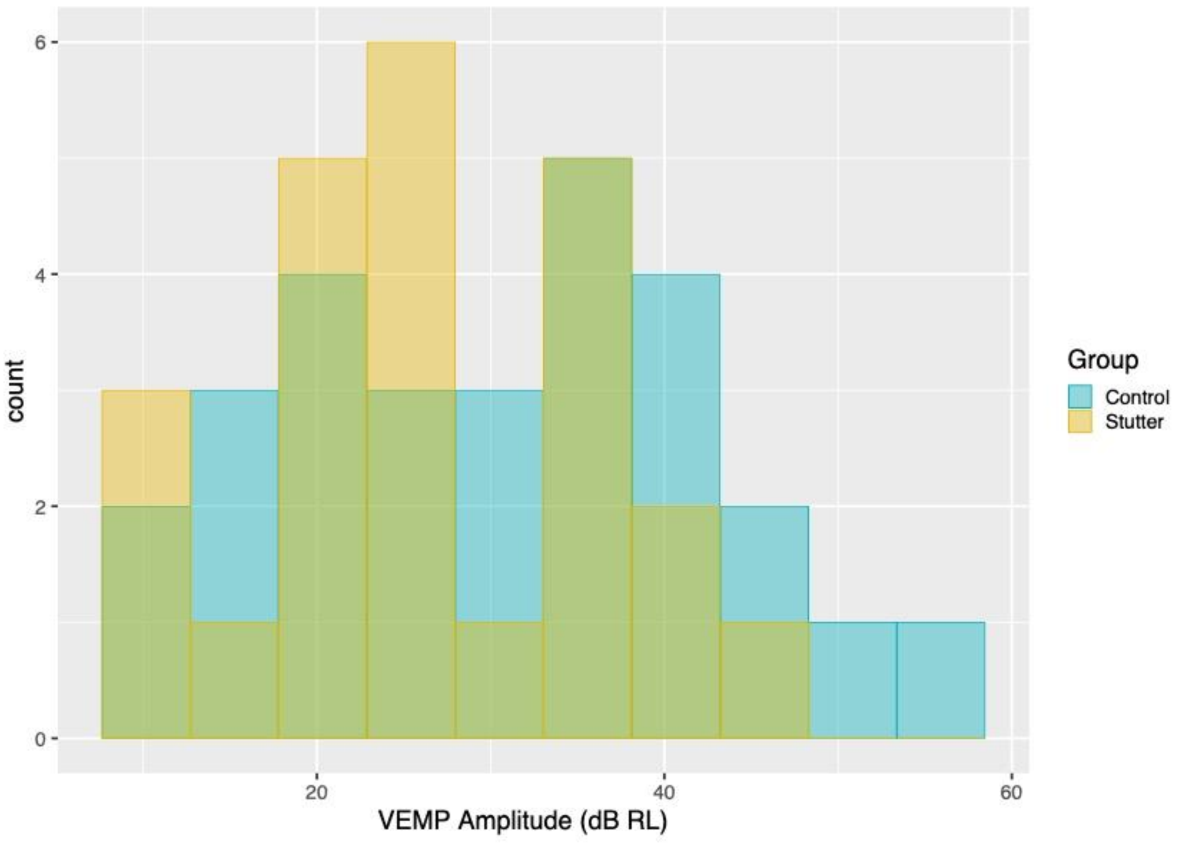

Figure 16: Histogram of VEMP p1-n1 amplitudes for PWS and paired controls for the pilot study (compare with figure 6). The histogram contains repeated measurements for two groups of 5 participants per group at different stimulus levels. As such, it suggests shape of

distribution, but is not appropriate for statistical comparison (statistical comparison is by linear mixed-effects regression modelling).

Individual participant slopes are shown in figure 18. Due to limitations of the pilot study (e.g. absence of the head bar and corresponding reduction in control over neck tension), it is unsurprising that the slopes are more variable than for data in the main study.

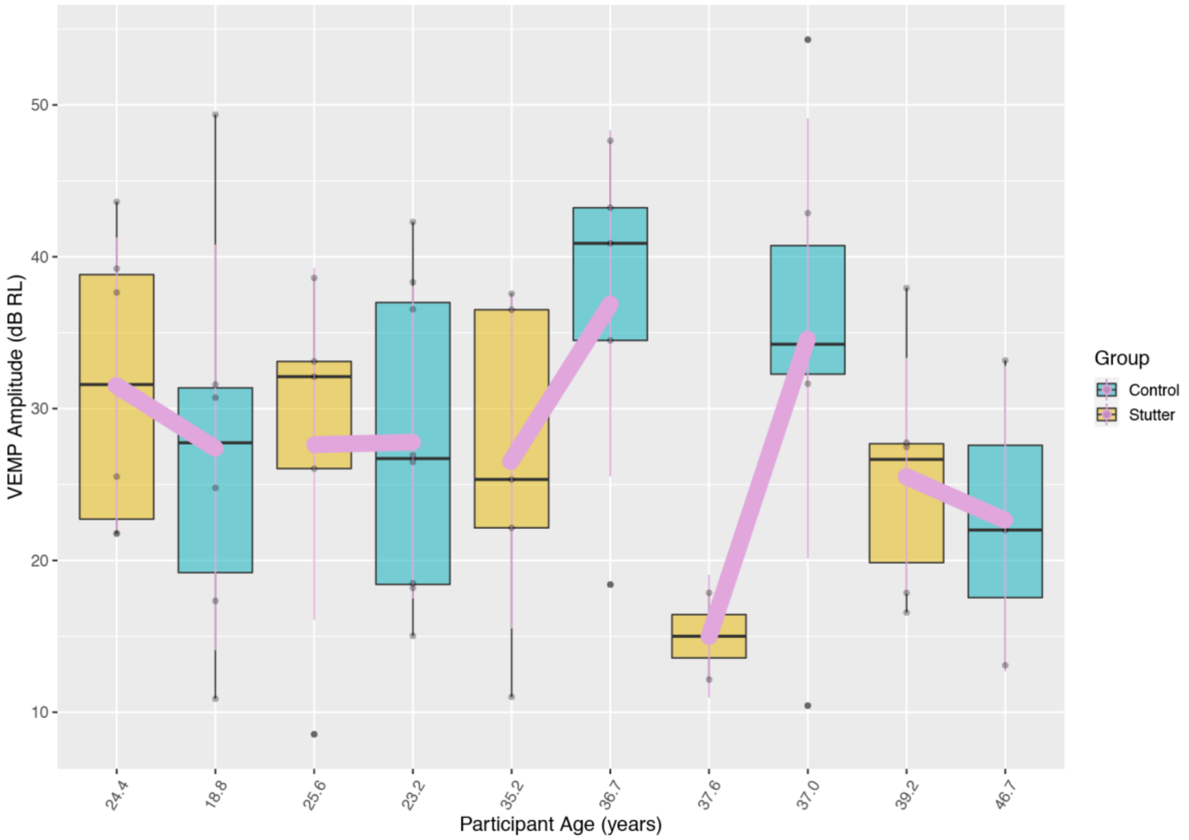

Figure 17: Box plots showing distribution of VEMP amplitudes per participant (across all participants in the pilot study), and participant age. Compare with Figure 6. Lines link the mean VEMP amplitudes of participants who stutter (PWS) with those of their paired controls. Pairing is less exact than for participants detailed in the main study.

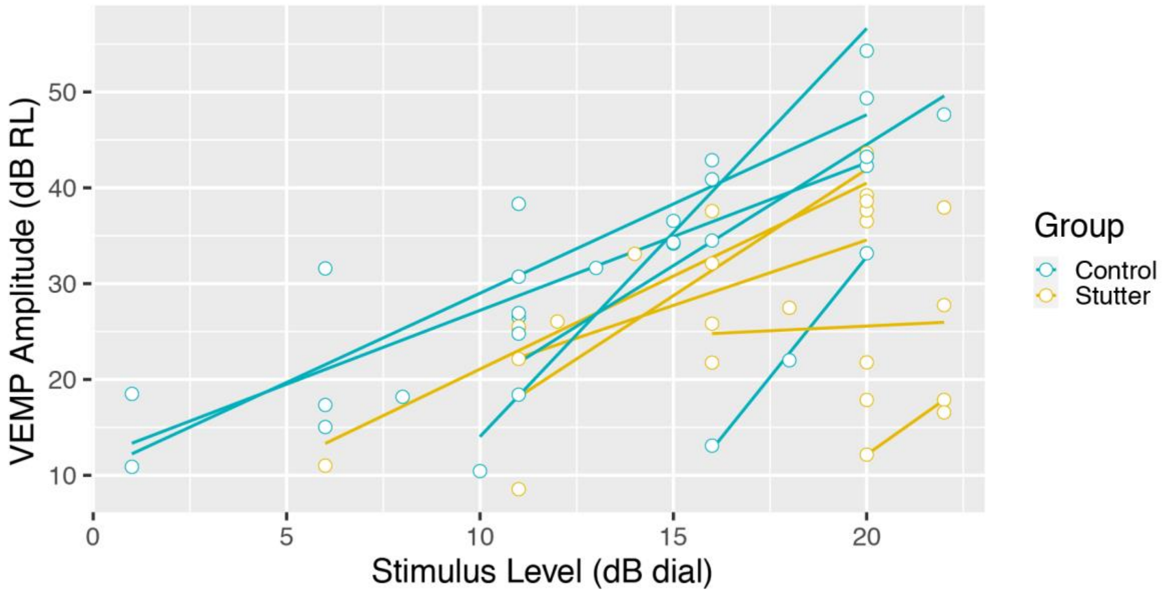

Figure 18: Per participant slopes of stimulus level (dB dial) versus VEMP amplitude (dB RL), showing participants who stutter (PWS) and paired controls. Compare with figure 10. Due to limitations of the pilot study slopes are more variable than the main study. Due to calibration differences stimulus levels are not comparable between main and pilot studies.

The final model for the pilot study is shown in figure 19.

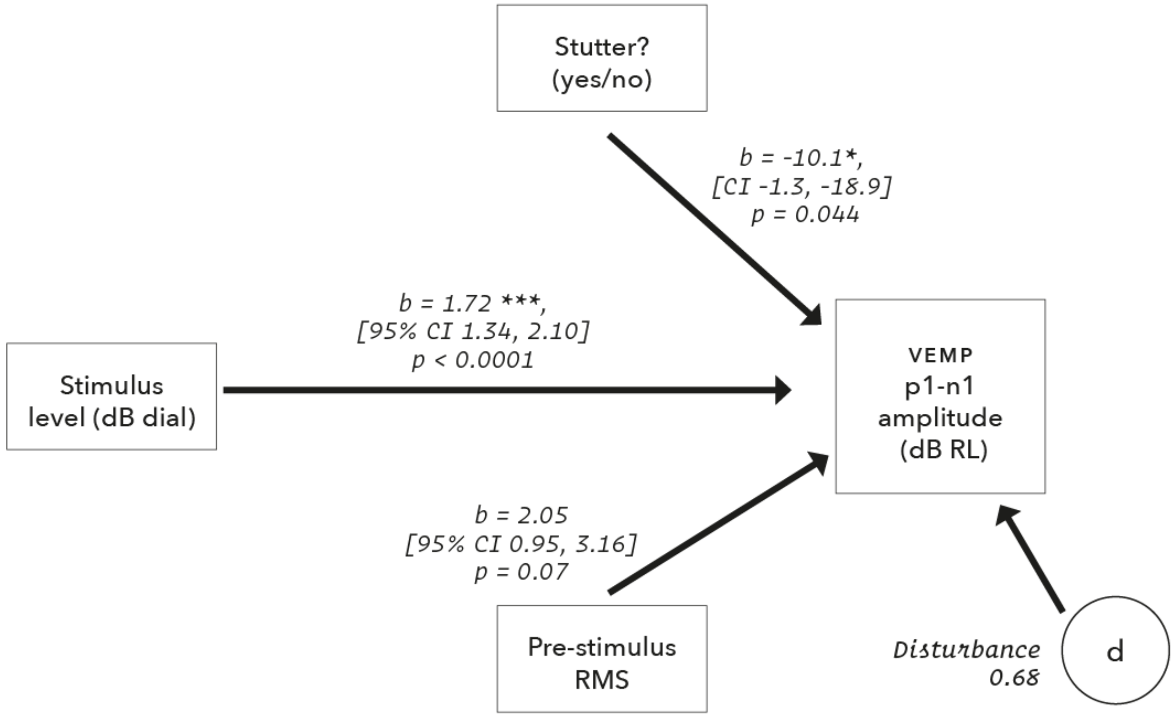

Figure 19: Final model for VEMP amplitude in the pilot study. Compare with figure 9.

No statistically significant group differences were found for peak to trough VEMP latency with pilot study data. Figure 20 shows latencies collected across all participants and all stimulus levels, including repeat measurements.

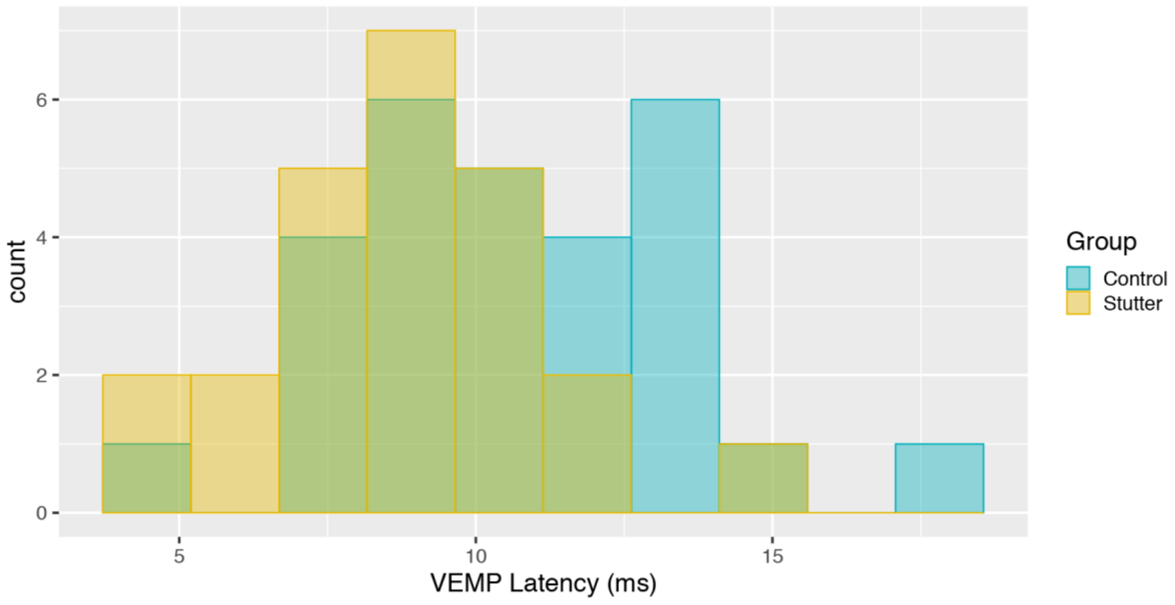

Figure 20: Histogram of latency difference between VEMP peaks and troughs (p1-n1) for the pilot study. The histogram contains repeated measurements for two groups of 5 participants per group. As such, it suggests shape of distribution and direction of group difference, but is not appropriate for statistical comparison (statistical comparison is by linear mixed modelling). Compare with figure 12.

Data appear normally distributed, with no indication of a group difference. Variation across participants with stimulus level is shown in figure 21. There is no statistically significant interaction. Pearson's correlation coefficient between VEMP latency and stimulus level is

$r\ (22) = -0.35$, $p = 0.09$, 95% CI [-0.66, 0.06] for PWS data
$r\ (26) = -0.30$, $p = 0.12$, 95% CI [-0.61, 0.08] for control data

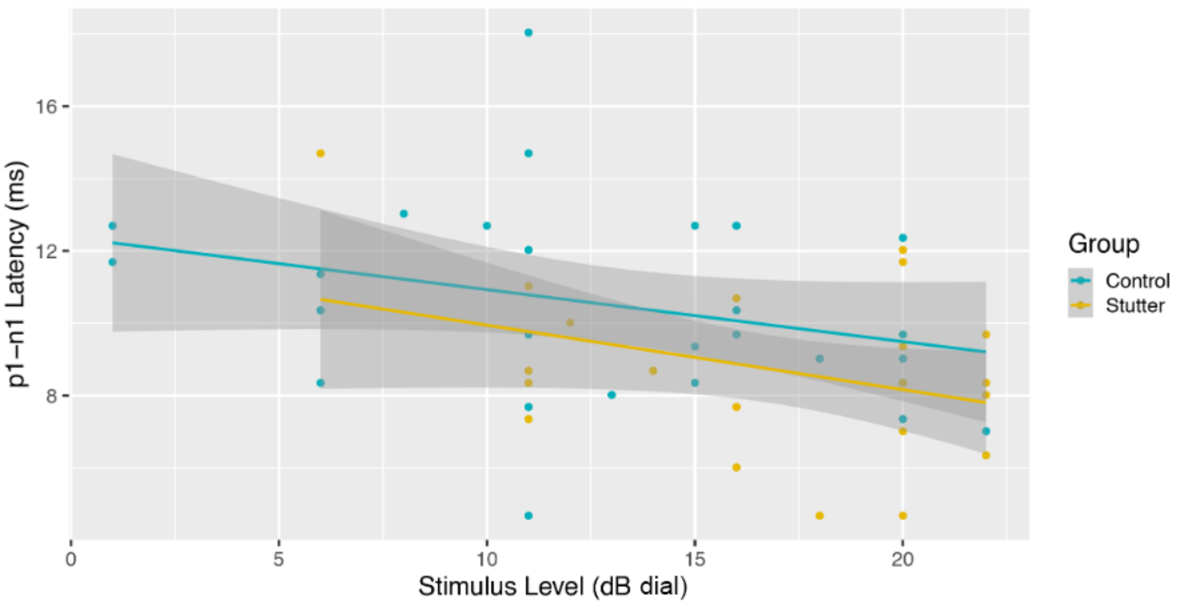

Figure 21: Variation of peak to trough latency with stimulus level for the pilot study. Compare with figure 13.

Group comparison was carried out through linear mixed model analysis, with *p* values generated by likelihood ratio comparisons between the following models:

```
model_null:  logp1n1 ~ 1   + (1|PiD)
model_diff:  logp1n1 ~ 1 + isPWS + (1|PiD)
```

There is no statistically significant difference between groups (chi squared (1) 2.6, *p* = 0.10).

4.4 Alternative linear mixed-model regression analyses for pilot study

Table 6 shows additional linear mixed models for the pilot data. The head bar was not available in the pilot study, so neck tension was generated via head raise from supine. Because of this, neck tension was expected to be more variable than in the main study. To account for the variability, models were tested with pre-stimulus background RMS tension (which corresponds to neck tension) as a covariate.

Model	Comments	**PWS-CONT diff** (std err)	**p-value** [(df) χ^2]
**Fixed slope** logp1n1 ~ dB + isPWS + (1|PiD)		**-10.3** (5.0)	**0.060** [(1) 3.52]
**Random slope** logp1n1 ~ dB + isPWS + (1 + dB|PiD)	Singular fit	**-6.7** (2.9)	**0.040*** [(1) 4.2]
**Fixed slope with RMS** logp1n1 ~ dB + RMS + isPWS + (1|PiD)	Effect of RMS is 2.1 (std err 1.1)	**-10.1** (4.5)	**0.044*** [(1) 4.05]
**Random slope with RMS** logp1n1 ~ dB + RMS + isPWS + (1 + dB|PiD)	Convergence Error; effect of RMS is 2.2 (std err 1.1)	**-6.8** (2.7)	**0.035*** [(1) 4.43]

Table 6: Linear mixed model comparison for data from the pilot study. Variables as are described in table 3, except for the addition of "RMS", which corresponds to neck tension. "RMS" is the normalised pre-stimulus background RMS EMG amplitude for each sequence of 300 stimulus presentations (see section 2.5 for details).

As anticipated, neck tension affected VEMP amplitude (b = 2.05, 95% [CI 0.95, 3.16], *p* = 0.07). The effect is large in comparison to data collected with a normative group of 48 (Gattie, Lieven & Kluk, in preparation) who used the head bar and had a pre-stimulus neck tension effect of b = 0.32 (95% CI [-0.07, 0.72], *p* = 0.11). For this reason, the model chosen has neck tension as an additional predictor. For consistency with the main study, a fixed slope model was chosen.
